# An Appraisal of the Classic Forest Succession Paradigm with the Shade Tolerance Index

**DOI:** 10.1371/journal.pone.0117138

**Published:** 2015-02-06

**Authors:** Jean Lienard, Ionut Florescu, Nikolay Strigul

**Affiliations:** 1 Department of Mathematics & School of Art and Sciences, Washington State University Vancouver, Washington, United States of America; 2 Financial Engineering Division and the Hanlon Financial Systems Lab, Stevens Institute of Technology, Hoboken, NJ, USA

## Abstract

In this paper we revisit the classic theory of forest succession that relates shade tolerance and species replacement and assess its validity to understand patch-mosaic patterns of forested ecosystems of the USA. We introduce a macroscopic parameter called the “shade tolerance index” and compare it to the classic continuum index in southern Wisconsin forests. We exemplify shade tolerance driven succession in White Pine-Eastern Hemlock forests using computer simulations and analyzing approximated chronosequence data from the USDA FIA forest inventory. We describe this parameter across the last 50 years in the ecoregions of mainland USA, and demonstrate that it does not correlate with the usual macroscopic characteristics of stand age, biomass, basal area, and biodiversity measures. We characterize the dynamics of shade tolerance index using transition matrices and delimit geographical areas based on the relevance of shade tolerance to explain forest succession. We conclude that shade tolerance driven succession is linked to climatic variables and can be considered as a primary driving factor of forest dynamics mostly in central-north and northeastern areas in the USA. Overall, the shade tolerance index constitutes a new quantitative approach that can be used to understand and predict succession of forested ecosystems and biogeographic patterns.

## Introduction

Forest succession is a continuous stochastic process that occurs at the level of individual trees and results in the replacement of one tree species by another at the level of forest stands [1–3]. Understanding the mechanisms underlying forest succession has remained a challenging scientific problem for more than a century [4–6]. Existing succession theories are mostly qualitative and represented as conceptual models having substantial limitations in their appraisal and validation [7–9]. Quantitative forest descriptions are typically based on simple indicators (e.g., time since the last major disturbance) or on macroscopic stand characteristics calculated using forest surveys (e.g. stand age and size structures, tree abundance, biodiversity, and biomass). However, these parameters are only remotely linked with forest succession. The lack of quantitative characteristics capable to quantitatively describe successional patterns in space and time hinders our ability to understand and predict changes of forested ecosystems. Ideally, we would like to have quantitative variables describing forest succession under different disturbance regimes. Such variables can be used to analyze stand dynamics and spatial forest patterns within already existing modeling frameworks, such as the hierarchical patch dynamics concept [10, 11].

The classic succession paradigm has been formulated based on observations of temperate forest patterns in Wisconsin, Michigan and New England [4, 12] and in northern and Central Europe [13]. In this type of forest the gap dynamics and shade tolerance driven succession are most noticeable and easy to observe. In a broad range of plant ecology literature, including in major textbooks, shade tolerance is considered as a primary factor underlying forest successional dynamics [14–16]. North-American tree species can also independently be classified as early and late successional species based on their life history and physiological traits [6, 17]. In the classic shade tolerance succession paradigm, species that are shade intolerant and tolerant are analogous to early and late successional species, respectively [18]. Replacement of early successional trees by late successional trees is driven by small scale disturbances caused by wind, tree diseases and tree removal [19]. Large scale disturbances such as hurricanes, severe forest fire, clearcutting and some rare catastrophic events e.g. volcano eruptions significantly change the successional stage of forest stands by promoting development of early successional species [20, 21]. Particular disturbances often have an intermediate scale and can be placed on a continuous scale of disturbances [22, 23]. The interplay of disturbance regimes and successional processes results in complicated and poorly understood multidimensional spatiotemporal patterns [23].

The goal of our research is to develop a quantitative approach that can be used to appraise succession of forested ecosystems. In the present work we introduce a macroscopic stand characteristic called the shade tolerance index defined as a weighted average of shade tolerant trees in a stand. We employ this new variable to describe patch-mosaic properties of the U.S. forests using the Forest Inventory and Analysis (FIA) Program data (http://www.fia.fs.fed.us/). This database has been collected by the U.S. Forest Service since the late 60s by surveying forested ecosystems across all U.S. ecological domains. We compare the shade tolerance index to the continuum index in southern Wisconsin forests using surveys from the FIA database, matching the location of the classic study of Curtis and McIntosh [24]. We also assess the relevance of shade tolerance index to understand forest succession in White Pine—Eastern Hemlock forest of northeastern US. This is done using two approaches: analysis of approximated chronosequence from the FIA database and individual-based computer simulations. We describe the shade tolerance index in mainland USA, and we demonstrate that it does not correlate with other macroscopic characteristics of forest stands. We finally characterize the dynamics of shade tolerance index based on re-sampled plots in the database and we sketch geographical areas based on the importance of shade tolerance as a driving force for forest succession. The appendices include description of the FIA database used in this study, quantitative tables of shade tolerance for North-American trees, and additional statistical results.

## Shade tolerance index and succession dynamics

### Shade tolerance and the classic model of succession

Shade tolerance is the ability of a tree to survive and develop under light limited conditions. Overall, tree species can be ranked by their tolerance to different factors affecting tree growth and survival, such as pollutants, heat, drought and nutrient deficiency. Therefore, shade tolerance can be thought of as just one of these tolerance types [25–27]. However, shade tolerance plays a special role in forest development since light is one of the most important factors for tree growth [25, 28, 29]. Ecological effects of shade are connected with all aspects of a plant life cycle including growth, reproduction, and mortality. This was noticed in numerous publications by both professional foresters and amateur naturalists. In particular, Pliny the Elder described some ecological effects of shade more than 2000 years ago (Chapter 18 of [30]. Henry David Thoreau wrote in his famous 1860 essay on forest tree succession: “The shade of a dense pine wood, is more unfavorable to the springing up of pines of the same species than of oaks within it, though the former may come up abundantly when the pines are cut, if there chance to be sound seed in the ground” [31]. The shade tolerance concept based on empirical data was developed in the XIX^th^ century and substantially influenced further development of plant succession theory in XX^th^ century [4, 7, 12]. In particular, as early as 1852, German forester Heyer [32] has published this conceptual framework and a shade tolerance ranking table of European trees. Extended studies of the late 1800s to early 1900s resulted in hundreds of research papers and several books providing synthesis and shade tolerance tables for tree species in the northern Hemisphere [13, 28, 33, 34].

The shade tolerance tables classify tree species by their shade tolerance patterns along a linear scale [28, 29, 35]. A typical shade tolerance classification consists of 5 uniformly distributed values: very intolerant, intolerant, intermediate, tolerant and very tolerant trees. The earlier classifications have been based on empirical observations of tree species growth under light limiting conditions, including leaf density, rate of death of the bottom tree branches shadowed by the upper tree branches (self-pruning), self-thinning of forest stands, density of trees in the understory and tree mortality [28, 33, 34]. Early experimental studies examined physiological and morphological mechanisms underlying shade tolerance, including differences in leaf morphological structure and photosynthetic activity to discover mechanisms of shade tolerance [28, 33, 34]. The modern species-specific shade tolerance tables have the same empirical character, but have been developed using the survey method. This is where teams of professional experts independently evaluate different plant species with respect to their shade tolerance and the final ranking is represented as a statistic of these independent evaluations [29, 36]. In particular, the shade tolerance tables for the US forests proposed by Zon and Graves in 1911 [28] were based on empirical observations and designed to be analogous with European trees, for which several different classifications had been previously established. These tables were revised for the first time 38 years later by Baker [29], who employed summarized opinions of 55 foresters as well as published studies. The next substantial revision came after another 50 years, when Humbert et al. [36] provided the shade tolerance values not only for tree species but also for shrub, herbaceous, bryophyte and lichen species. These consequent revisions obtained with the survey methods expanded the range of species covered and have overall confirmed the earlier ranking of the major US tree species proposed by Zon and Graves [28]. These existing shade tolerance rankings are species-level expectations of individual-level random variables, and it is broadly recognized that there exists substantial intraspecific variability of shade tolerance depending on the environmental conditions and tree age [28, 29, 35–37]. At the same time numerous quantitative studies based on the species-level shade tolerance values demonstrate that shade tolerance is one of the major physiological traits correlated with other traits such as understory mortality and growth [15, 35, 38, 39], photosynthetic and allocation patterns [40], tree morphology [41], crown ratio [42], root distribution [43] and seed size [44].

The concept of shade tolerance is naturally linked with the concepts of forest succession and disturbance. The classic successional model employs shade tolerance to explain the following transitions in canopy tree composition after a major disturbance [3, 16, 45]:

$$\begin{array}{c} \text{primary successional trees (very shade intolerant)}\\ ↓\\ \text{early successional trees (shade intolerant)}\\ ↓\\ \text{late successional trees (shade tolerant)} \end{array}$$

This conceptual model historically states that the initial canopy is formed mostly by primary and early successional species that are shade intolerant but tolerant to numerous adverse factors affecting tree growth in the absence of the microclimate created by forest canopy, for example water limitation, extreme temperatures, ice damage [13, 33, 46, 47]. After the canopy is closed, forest succession is driven by individual tree mortality and small disturbances that develop relatively small gaps approximately equal to one average crown area which facilitate recruitment of shade tolerant understory trees [5, 16, 45, 48, 49]. The conceptual model of the forest gap dynamics [2, 3] includes two processes: 1) the development of forest gaps in the canopy after the death of large trees, and 2) recruitment of understory trees into the canopy. The gap facilitates recruitment of understory trees which are mostly shade tolerant [19]. Intermediate and large scale disturbances can create large canopy openings and promote development and recruitment of faster growing early successional tree species, which are mostly shade intolerant [9, 22].

### The shade tolerance index

According to the classic paradigm described in the previous section, the proportion of shade tolerant versus intolerant trees is linked to the forest succession stage. We propose a quantitative parameter, the *shade tolerance index*, *δ*, to characterize *stand successional stages* as follows:

1. Shade tolerance of every *tree species* is quantified by a number from an interval *ρ* = [0, 1] where the range spans very intolerant to tolerant species. We will call the number *ρ* the *shade tolerance rank of a tree species*. Specifically, we quantify the species as following: very intolerant = 0, intolerant = 0.25, intermediate = 0.5, tolerant = 0.75, and very tolerant = 1, according to [29, 36, 50] (see S1 Appendix for computation details and S6 Appendix for species rankings).
2. The shade tolerance index of the stand, *δ*, is defined as a weighted sum of the species abundance on their shade tolerance ranks defined as:
  $$\delta =\sum_{j=1}^{k}\rho_{j}\alpha_{j}.$$ (1)
  In this formula *α*_*j*_ is a measure of relative abundance of a species *j* in the stand, *ρ*_*j*_ is a shade tolerance rank of the species *j*, and the index *j* runs through all *k* tree species present in the stand.

The relative abundance parameter *α*_*j*_ is estimated using the formula:

$$\alpha_{j}=\frac{\Omega_{j}}{\sum_{i=1}^{k}\Omega_{i}},$$ (2)

where Ω_*j*_ is a measure of abundance of the tree species in the stand. *α*_*j*_, is a number in the interval [0, 1] and, obviously, $\sum_{j=1}^{k}\alpha_{j}=1$ regardless of the choice for the measure Ω_*j*_. Since all *ρ*_*i*_ are in [0, 1], the shade tolerance index *δ*, is also a number from [0, 1]. Specifically, *δ* is equal to 0 if all the trees in the stand are very shade intolerant and equal to 1 if all trees are shade tolerant.

In S1 Appendix, we address the technical points of the practical calculation of Ω_*j*_ as well as *ρ*_*j*_. We also investigate the robustness of the *shade tolerance index of the stand*, *δ*, with respect to the way in which Ω_*j*_ and *ρ*_*j*_ are defined. In particular, Ω_*j*_ was computed with: (a) the number of trees of species *j*, (b) their basal area, and (c) their biomass. Our results demonstrate that these estimates are correlated and in the results presented in this work Ω_*j*_ is based on the basal areas of alive trees. Ω_*j*_ could also be calculated using some particular group of trees, for example only canopy or understory trees.

The shade tolerance index can be calculated at the level of individual trees with *ρ* as a function of tree life history, size, age and environmental factors, however, given the lack of such information, we employ the average tree species values according to the shade tolerance ranking tables.

### Comparison with the forest continuum index

The classic forest succession paradigm was historically developed under a strong influence of the studies conducted in the Lake States (MI, WI and MN). In particular, both Clements [4] and Cowles [12], the founders of this paradigm, have done their field research in this area. Later, Curtis and McIntosh [24] have developed the classic continuum index to describe successional patterns in southern Wisconsin. This landmark paper was an important step in the development of forest succession theory that influenced numerous studies and theoretical developments, in particular the continuum hypothesis of vegetation [51, 52], gradient analysis of vegetation [53] and ordination of plant communities [54–56]. We anticipate that the shade tolerance index is capable of capturing successional patterns, and we compare these indexes in southern Wisconsin.

Curtis and McIntosh [24] studied successional patterns in a collection of 95 selected forest stands. The authors selected this snapshot of the forest stands at different successional stages to characterize the continuous transition from early successional stages to the climax stage. The importance values corresponding to tree species have been computed based on respective species dominance within the stands. Using tree dominance data, these stands were positioned along the continuum line representing a forest succession sequence. According to this continuum index axis, Curtis and McIntosh [24] assigned numerical values to the tree species within the stands called the climax adaptation numbers. The authors have also demonstrated that the constructed continuum index is related to soil factors such as exchangeable calcium concentration and soil acidity.

Curtis and McIntosh [24] did not employ the concepts of shade tolerance and gap dynamics to derive the climax adaptation numbers. However, they indicated that the calculated climax adaptation numbers are parallel to the shade tolerance ranking used in forestry ([24], pp. 489–490). Our mechanistically-based approach of obtaining the successional axis is substantially different from the empirical-based approach employed by Curtis and McIntosh [24]. In particular, the shade tolerance index is based on independently established shade tolerance rankings, while the continuum index relies solely on the composition of sampled plots.

**Fig 1:**
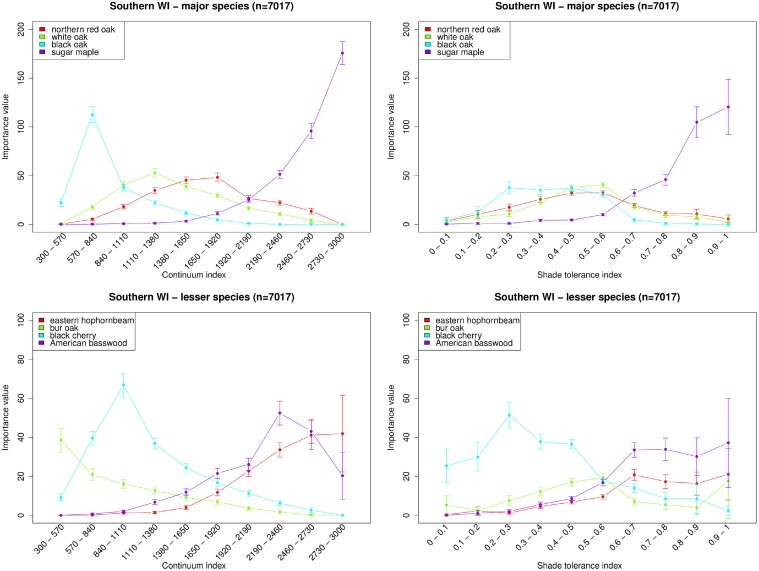
Comparison of the continuum index (*left*) and shade tolerance index (*right*) in southern Wisconsin. Similarly to the original study [24], the species were split in two groups: major (*top*) and lesser (*bottom*) species. Bars indicate the standard error of the mean.

The original data used by [24] is not available, and we reproduced a similar analysis using the FIA data. We have calculated the same statistical characteristics using a sample of 7017 FIA plots on mesic soils corresponding to the same geographic area as the original article by using Figure 1 in [24] as a reference (see Figure 1 in S2 Appendix). The original study took into account only forest stands that were: 1) “natural forests (i.e. not artificially planted)” of a minimum size of 40 acres, 2) “free from disturbances in the form of fire, grazing or excessive cutting”, and 3) “upland land forms on which run-off water never accumulate” ([24], p. 480). Similarly to the original study, we restricted the analysis to mesic soils (criterion 3). However, in contrast with [24] we analyzed all forested plots, including planted forests (criterion 1), and we did not filter out plots based on environmental disturbances (criterion 2).

Our extension of [24] analysis to a wider range of plots resulted in a more diverse species composition. Indeed, only 21 species were considered in the original study, while the actual database plots that we used contain 84 species. To be able to compute the original importance values [24], we only consider the trees belonging to the 21 species in the original work when computing both the continuum and the shade tolerance index. This modification led to a smaller magnitude of the importance value, but overall our results are in good agreement with the original results (Fig. 1 vs Figures 5, 6, 7 in [24]). Our results are also in agreement with the work of Rogers et al. [57] who re-sampled the same sites as Curtis and McIntosh [24] some 50 years later. In particular, we notice that the relative importance value of red oak (*Quercus rubra*), black oak (*Quercus velutina*) and secondarily white oak (*Quercus alba*) have decreased compared to sugar maple (*Acer saccharum*).

**Fig 2:**
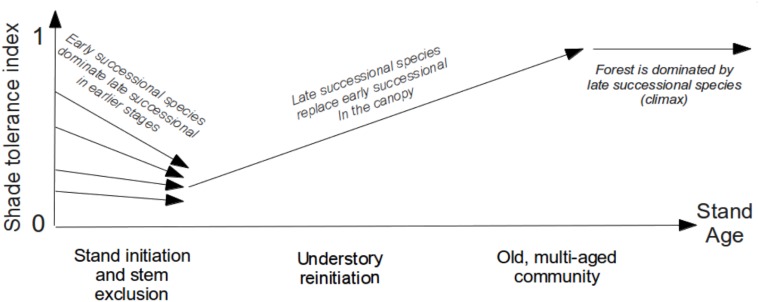
Conceptual model of shade tolerance index dynamics after a major disturbance. The initial proportion of seedling of shade tolerant and intolerant species can be very different due to the nature of the disturbance and biological legacies. Shade intolerant species outgrown shade tolerant species during the stem exclusion stage of the forest stand development, leading to the index decrease. Shade tolerant species gain an edge over shade intolerant species via canopy gap dynamics, resulting in the index increase during the understory reinitiation stage, and its further stabilization as the forest approaches the old growth stage.

**Fig 3:**
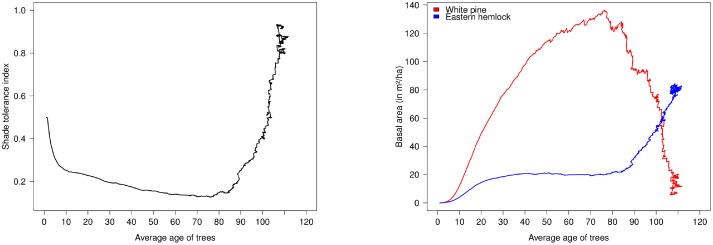
Computer simulation of White Pine—Eastern Hemlock forest stand. Shade tolerance index as a function of the implied stand age (left) and cumulative basal area of White Pine and Eastern Hemlock (right) as a function of the implied stand age. The simulation is conducted using the crown plastic SORTIE model following [62] for one hectare and 1000 years, the parameter values are similar to the original paper except the mortality of both species was reduced to 5%.

**Fig 4:**
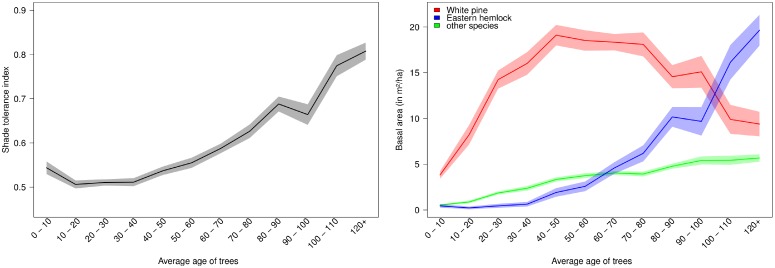
Approximated chronosequence of White Pine—Eastern Hemlock forest stands in the northeastern US. Analyzed data consist of 1375 USDA FIA plots, where these species account for more than 75% of the total basal area (S3 Appendix). Shade tolerance index (left) and cumulative basal area of White Pine and Eastern Hemlock (right) as a function of the implied stand age (S1 Appendix). The shaded areas represent the standard error of the mean. The last bin “120+” includes 3% of the plots.

**Fig 5:**
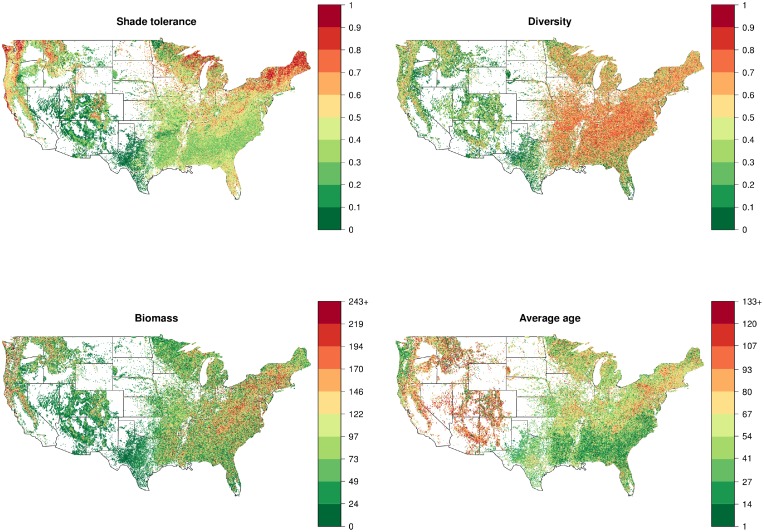
The stand-level characteristics of plots for all years demonstrate very heterogeneous forest types in the US, with no obvious common distribution pattern between indicators. Maps of subsets of plots from different decades are similar and can be found in S1 Appendix.

Overall, our analysis shows that the successional pattern observed by Curtis and McIntosh [24] did not change substantially over the several decades, and that the original plot sampling restrictions did not substantially affect these patterns. Furthermore, our analysis of a large number of plots from the FIA database allows us to observe successional patterns on a broader range of the continuum index than Curtis and McIntosh [24] (from 300 to 3000 in Fig. 1 and only from 632 to 2650 in the original paper). There are some remarkable successional patterns observed on these additional intervals of the continuum index scale ([300–700] and [2600–3000]). In particular, black oak has an obvious peak of its importance value at about 700 (Fig. 1), that was not observed in the original study and the sugar maple’s importance value monotonically increases along the full spectrum of continuum index. There is an overall good qualitative correspondence of each species ordination between the continuum index axis and the shade tolerance axis (Fig. 1), demonstrating the ability of the shade tolerance index to describe forest succession. Other traditional indicators such as biomass, basal area, biodiversity or stand age do not allow for discrimination between species (Figures 2 and 3 in S2 Appendix). We assessed quantitatively the overall similarity between the continuum index and the shade tolerance by computing the Pearson’s correlation coefficient *r* between these two variables, regardless of the species compositions of plots. With a value of *r* = 0.57 in the 95% confidence interval [0.55, 0.59], these two variables display a medium to strong correlation (Figures 4 and 5 in S2 Appendix).

### Successional dynamics

The forest stand dynamics theory states that after a major disturbance stand development follows four consecutive stages: initiation, stem exclusion, understory reinitiation, and old-growth [16, 47]. The *stand initiation stage* marks the onset of succession by regeneration of open space from seed, sprouts and advance regeneration, and lasts until the canopy closes. Different disturbances leave various types of biological legacies providing highly variable initial species composition [58, 59]. In the second stage of *stem exclusion*, the light-driven competition becomes the major determinant of survival, resulting in a domination of fast-growing early successional species. The third stage, *understory reinitiation*, is characterized by the selective recruitment of understory trees in the canopy through gap-dynamics. The final stage, the *old-growth* corresponds to the climax state of the forest, where the species composition is stable.

The shade tolerance index will be used to interpret and quantitatively measure these dynamics. Fig. 2 summarizes the conceptual model of shade tolerance driven successional dynamics after a major disturbance. From the definition of the shade tolerance index, we expect an initial decrease of the index value during the reinitiation and stem exclusion stages. This is due to the higher mortality of shade tolerant species that are less resistant to the large number of unfavorable environmental factors, as well as to their exclusion by faster growing shade intolerant species. During the understory reinitiation stage, the shade tolerance index will monotonically increase due to recruitment of shade tolerant species in the canopy. In the old community stage we expect the shade tolerance index to remain stable in the absence of intermediate and large scale disturbances. This conceptual model is in accordance with our results, including analysis of the White Pine—Eastern Hemlock succession presented below and data mining of FIA dataset (see the following section and S3 Appendix).

We assess the conceptual model of Fig. 2 by considering a two-species system consisting of shade intolerant White Pine and shade tolerant Eastern Hemlock. This system embeds the classic shade tolerance paradigm as it demonstrates the shade tolerance trade-off, and at the same time, it plays an important role in the successional dynamics of the temperate forest in the Eastern part of the United States [50, 60, 61]. We analyze the predictions of a forest gap model [62], and compare them to the approximated chronosequence extracted from the FIA database, in order to examine the shade tolerance dynamics on this bicultural system.

Traditionally, individual-based gap dynamics models are employed to quantitatively predict stand development after major disturbances [46, 63, 64]. Gap models are parameterized by individual-tree data and can be used as a tool to simulate shade tolerance driven succession and statistical characteristics of canopy recruitment [65, 66]. The White Pine—Eastern Hemlock system was previously simulated using the crown plastic version of the SORTIE model ([62] Figure 12, p. 535), and this model is employed now to assess the temporal dynamics of shade tolerance index. In this model, the shade tolerance trade-off is simplified and limited only to differences in tree growth and mortality, and does not involve interspecific differences in the seed dispersion and reproduction strategy. In particular, both species have the same initial number of seedlings and new seedlings emerge every year in the same quantity. This simulated scenario of the forest succession has been started after a major disturbance, and Fig. 3 shows changes of shade tolerance index. The successional dynamics are essentially driven by the shade tolerance trade-off: White Pine is the large tree that grows faster in the full light, but has a higher mortality in the understory, while Eastern Hemlock grows slower but survives in the light limited conditions. The canopy gaps development is randomly driven by individual tree mortality that provides lottery recruitment of understory trees, and this mechanism is sufficient to obtain the early decrease followed by a progressive increase of shade tolerance postulated by the conceptual model of Fig. 2.

We compare the results of computer simulations with the statistical analysis of White Pine—Eastern Hemlock forest stands from the FIA database (Fig. 4). We consider here approximate chronosequences, as the plots are ordinated relatively to the average age of trees (see S1 Appendix), the time since last disturbance being not available. The approximated chronosequence based on the FIA data can be used for model validation in case forest succession data obtained at one particular location are not available [67]. The stands are observed throughout northeastern parts of the US. We isolated all plots in the database with more than 75% of cumulative basal area composed by these two species, resulting in a pool of 1375 plots (see Figure 1 in S3 Appendix for their locations). The comparison of Figs. 4 and 3 demonstrates striking qualitative similarities between computer simulations and the chronosequence. Specifically, the initial distribution of seedling shade tolerance index decreases in the early years as the faster growing pioneering species start to dominate the canopy. The shade tolerance index then reaches high values as shade tolerant species eventually dominate the early successional species. Similarly, the cumulative basal areas show the same pattern of early dominance of White Pine before Eastern Hemlock take over in old stands. These trends observed both in FIA database and in the computer simulations reflect the conceptual model of Fig. 2.

## Spatiotemporal patterns of US forests

In this section we analyze forest stand mosaic in mainland US using the FIA dataset. The goal is to understand statistical relationships between forest characteristics and patch mosaic patterns related to shade tolerance. We first conduct a descriptive statistical analysis of shade tolerance patterns, and compare them to indicators of implied stand age, diversity, biomass and basal area. We then study shade tolerance dynamics by grouping all permanent plots according to the Bailey’s ecoregions classification, which relates climatic factors and species compositions (c.f. [68]; see Figure 1 in S1 Appendix for a map of the ecoregions). Our analysis allows us to delimit areas and conditions in which shade tolerance is a major driver for succession.

### Descriptive statistics and correlations

The stand-level characteristics display different spatial patterns in their distribution across regions (Fig. 5). To further study the spatial distribution of the characteristics, we classified plots according to geographically defined areas sharing common ecological properties. We employed Bailey’s province subdivisions because they are fine enough to discriminate between major plan formations, yet large enough to allow for the statistical description of forest patch mosaic (see Figure 1 in S1 Appendix). Descriptive statistics further confirm important disparities in the various indicator’s distributions across provinces (S1 Appendix).

**Fig 6:**
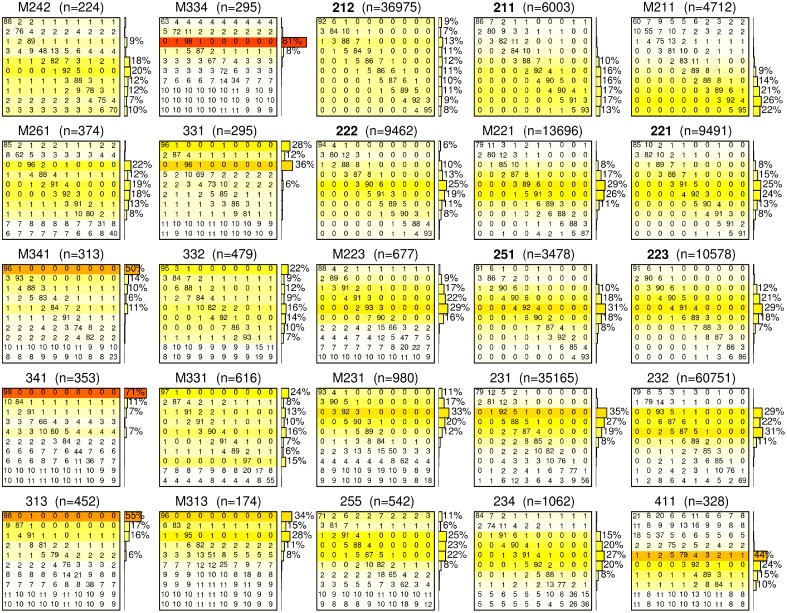
Representations for each ecoregion with *n* > 150 re-sampled plots of: -the shade tolerance index transition matrices (tables); -the current distribution of states (bar graphs). In each transition matrix, the value located in row *i* and column *j* is the probability of transition from state *i* to state *j* after 3 years (expressed in percents). The current distribution of the shade tolerance index is important to interpret these matrices, as transitions affecting the most numerous shade tolerance states have the most influence on future distributions. We indicate these distributions with a bar graph to the right of each matrix, and report it as colored rows in the matrices for easier reading (from white to red, for sparsely to largely represented shade tolerance index states). Placements have been chosen to approximately reflect geography, with northeastern ecoregions located in the top-right corner of this figure.

The analysis of correlation patterns indicates that the shade tolerance index displays weak correlations in the range of 0.12–0.26 with the other macroscopic characteristics studied: biomass, basal area, Gini-Simpson diversity index, species richness and average age of trees (Figure 3 in S4 Appendix). However, some of the other measures are correlated: biomass with basal area (confirming the previous study by Strigul et al. [11]), and Gini-Simpson diversity with species richness. Correlation matrices have been further calculated separately for all provinces in mainland USA (Figure 6 in S4 Appendix) and all inventory years with more than 500 plots recorded from 1968 to 2012 (Figure 5 in S4 Appendix). Correlations between different variables were virtually identical for all the inventory years and different ecoregions. This result is similar to what we obtained by analyzing another dataset for Eastern Canada forests [69]. The fact that shade tolerance index has been repeatedly shown to be uncorrelated with other macroscopic characteristics demonstrates its usefulness in the statistical description of the mosaic of forest patches.

### Ecoregion classification

To understand the shade tolerance dynamics, we modeled the shade tolerance index as a stochastic Markov chain process. We computed and analyzed the transition matrices for shade tolerance index by employing the original Bayesian approach developed in [69]. We summarize here the key points of our approach, and redirect to S5 Appendix for additional details. Firstly, we discretized the continuous [0, 1] range of shade tolerance index into 10 even states, the first state being [0, 0.1], and the tenth being [0.9, 1]. We then estimated a 3-year transition probability matrix in each province. Gibbs sampling is well suited to infer transition matrices, as it is by design robust to outliers (e.g. measurement errors in the field); however due its stochastic nature, transition matrices computed from a very small number of measurements are meaningless. In this study we consider the transition matrices obtained in provinces with more than 150 remeasured plots (Fig. 6).

The complexity of forest disturbance regimes leads to the nonmonotonic successional dynamics within stands, which is reflected in the non-zero coefficients of transition matrices (Fig. 6). Intermediate and large scale disturbances can promote development of shade tolerant or intolerant trees depending on stand conditions. These disturbances thus cause nonmonotonic behavior (such as sudden jumps and drops) of the shade tolerance index. In particular, in the case of a substantial part of the shade intolerant canopy trees being destroyed and the well-developed shade tolerant understory trees recruited into the canopy, we will observe the jump of the index. In another scenario, an intermediate or large disturbance of shade tolerant tree canopy with no developed understory will facilitate development of shade intolerant species and lead to a drop in the index. These transitions are captured in the matrices of Fig. 6.

Ecoregions with succession driven by shade tolerance should have transition probabilities in accordance with the conceptual model of Fig. 2, which predicts three different phases of shade tolerance dynamics: (a) a decrease in the early stages, followed by (b) an increase and eventually (c) a stabilization. The matrices of Fig. 6 capture all forest transitions disregarding the implied stand age. It is possible to study specifically the initial dynamics of shade tolerance index by computing transition matrices on the subset of plots with a stand age less than 20 years. These matrices (Figure 2 in S5 Appendix) demonstrate that a decreasing pattern in the early stages is quite common in the different ecoregions and that the shade tolerance index varies substantially within young forest stands in agreement with the conceptual model. The observed compositions of earlier successional forests are also in agreement with the stand dynamics theory stating that both earlier and late successional species occur during the stand initiation (Chapter 7, Figure 7.1 of [16, 59]]. However, the limited number of observed transitions of very young stands prevents this analysis to be applied on most US ecoregions. To study systematically the applicability of the shade tolerance succession paradigm to different ecoregions, we further derived two quantitative criteria based on (a) distributions of the shade tolerance index in all plots, and (b) transition matrices in all re-sampled plots (Fig. 6).

**Fig 7:**
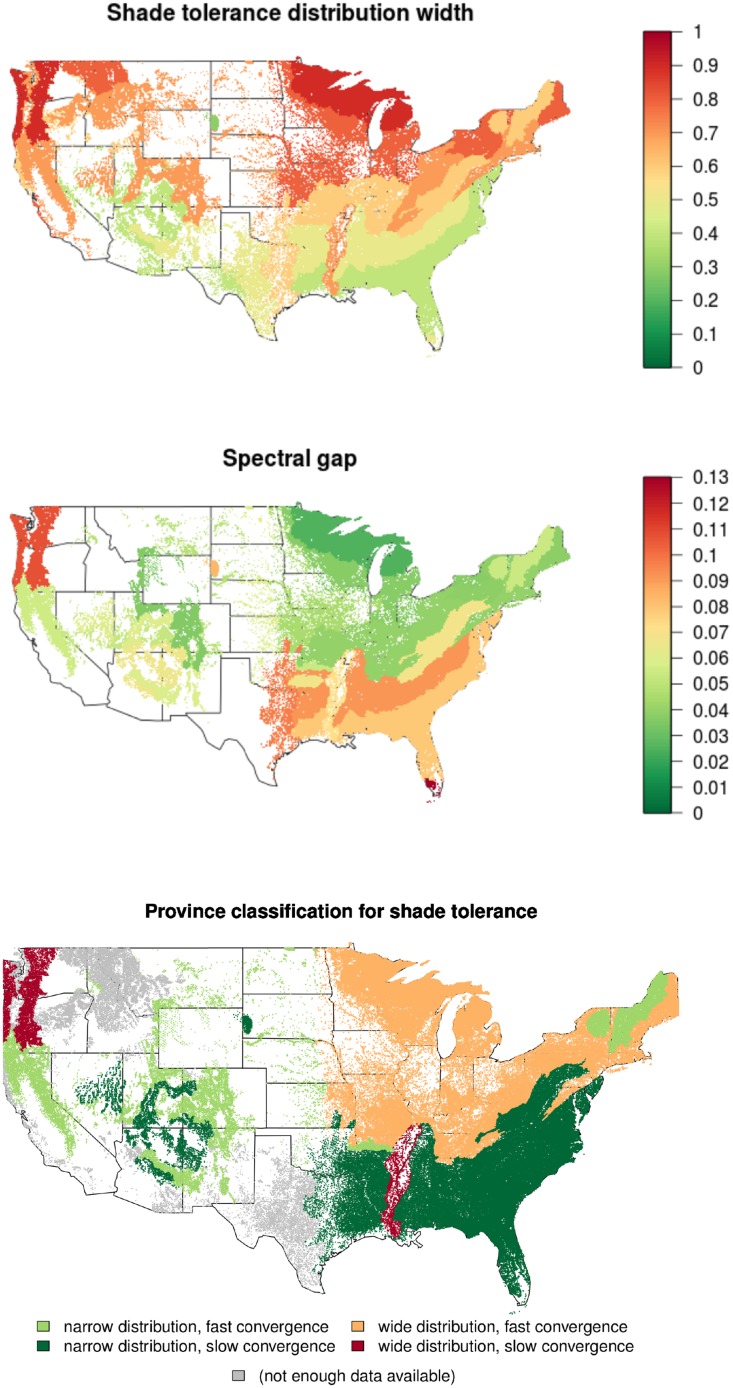
Classification of ecoregions based on shade tolerance distribution width and spectral gap. *Top:* the disparity of shade tolerance is measured as the length of interval encompassing 95% of the distribution. *Middle:* the spectral gap of the transition matrices quantifies the convergence rate after a disturbance, where low values mean that one plot is quick to reach equilibrium. *Bottom:* classification of ecoregions. Provinces where forest succession is primarily driven by shade tolerance exhibit a wide distribution of shade tolerance index as well as a fast convergence toward equilibrium.

First, the shade tolerance index should take substantially different values depending on stand age according to the conceptual model of Fig. 2. Indeed, projecting this model on forest stand mosaic within an ecoregion, we anticipate that stands with all possible shade tolerance index will occur, from low values (for plots in the early stages) to high values (for old-growth plots). Quantitatively, this disparity can be assessed by characterizing the width of the shade tolerance distribution in the forest inventory dataset. In particular, the limited width of the distribution indicates that the shade tolerance is not the primary factor in succession within a given ecoregion. We use the width encompassing 95% of the shade tolerance index as the first criterion for ecoregion classification (Fig. 7). The restriction of the distribution width to 95% removes outliers existing in the database that can be seen in distributions of all stand characteristics presented in S1 Appendix.

Second, we compute the spectral gap as a global measure characterizing the average time needed by a plot to reach the shade tolerance equilibrium after a random perturbation. Formally, the spectral gap is defined as 1 − *λ*_2_, where *λ*_2_ is the second highest eigenvalue of the transition matrix [70]. For a province, high values of this metric mean that the time needed to reach shade tolerance equilibrium is long, which could be interpreted either as a weak shade tolerance drive for succession or possibly as a longer canopy turn-over. We computed this metric only for provinces for which we can have a reliable estimate of the transition matrices (Fig. 7 in main text and Table 1 in S5 Appendix).

In the ecoregion classification, we characterize the shade tolerance distributions as either “wide” or “narrow” according to their value compared to the median distribution width (Table 1 in S5 Appendix). Similarly, we distinguish between “fast” and “slow” convergence toward equilibrium based on the relative spectral gap value compared to the median spectral gap. We were thus able to delimit different kinds of ecoregions based on their shade tolerance dynamics (Fig. 7).

We can distinguish four main types of ecoregion based on these two criteria. Some ecoregions exhibit “narrow” shade tolerance distributions, regardless of their rate of convergence toward equilibrium (light and dark green in Fig. 7). These regions are not good candidates for shade tolerance driven succession, as they lack the expected spatial mosaic of plots having different shade tolerance. Their rate of convergence toward shade tolerance equilibrium is difficult to interpret, because the dynamics of shade tolerance are primarily constrained by their narrow width. All ecoregions in the Dry Domain belong to this category, and they are either deserts and plain areas where succession mechanisms do not apply at all, or forested areas where the succession is strongly driven by water limitation and climatic conditions. Apart from the Dry Domain, only mountained areas belong in this category, and these usually display shade tolerance patterns directly linked to altitude, suggesting here also a weak or non-existent influence of shade tolerance for succession. Two ecoregions display a “wide” distribution of shade tolerance with a “slow” convergence toward equilibrium (red in Fig. 7). Displaying a mosaic of different shade tolerance across the plots, their succession should be driven by shade tolerance to some extent. However, their shade tolerance dynamics are slow, indicating (a) an existing but secondary drive of shade tolerance, or possibly (b) a strong influence of shade tolerance for succession but overall slow successional dynamics, related to a longer longevity of species and thus a slower turn-over of canopy trees. The remaining ecoregions have a “wide” distribution and a “fast” convergence of shade tolerance (orange in Fig. 7). These ecoregions associate both a spatial mosaic of patches with different shade tolerance index and relatively fast dynamics back to shade tolerance equilibrium after a random perturbation. They thus constitute the most suitable provinces to embed the shade tolerance based theory of succession, and are all clustered in north-central and northeastern parts of the US.

We have applied our shade tolerance classification at the level of the provinces (Fig. 7), however the classification provides clear distinctions at higher levels of the biogeographic hierarchy, i.e. at the division and domain levels (Figure 2 in S4 Appendix; [68]). This constitutes evidence that shade tolerance driven succession is strictly linked with large scale climatic factors and biogeography. Indeed, the biogeographic division of the US forests into domains, divisions and provinces is based on different principles than the shade tolerance based classification [68, 71]. The shade tolerance classification also explains some relationships between spatial distributions of different stand-level characteristics presented in Fig. 5. One immediate observation is the clear transitional patterns within the Humid Temperate Domain (200) of Eastern US. Indeed, forests in the Warm and Hot Continental Divisions (210, 220) are driven by shade tolerance succession (Fig. 7) and consist of highly diverse forest stand mosaics with respect to biomass, biodiversity and stand age distributions (Fig. 5, S1 Appendix). On the other hand, forests in the Subtropical Division (230) are not driven by shade tolerance succession and consist mostly of shade intolerant young stands differing in biomass (Fig. 5). The Lower Mississippi Riverine Forest Province (234) is an obvious exception in this division, as this truly unique province is located in Mississippi river floodplain and consists of low forested terraces and swamps [68].

Mountain provinces are typically distinct in their classification from surrounding provinces. In particular, M211 (Adirondack—New England Mixed Forest—Coniferous Forest—Alpine Meadow Province) and M221 (Central Appalachian Broadleaf Forest—Coniferous Forest—Meadow Province) are classified as not being driven by shade tolerance whereas other northeastern ecoregions are classified as being driven by shade tolerance. The vegetation of these two mountain areas is specifically determined by vertical zonation [68], where very close patches may be affected by very different climatic conditions, indicating the prevalence of other tolerances over shade tolerance. In addition, mountain forests have different understory light regimes and recruitment patterns, for example, a substantial fraction of mountain forests in New England (M211) is completely dominated by conifer species ranked as shade tolerant. Overall, mountain forests in New England and several other mountain areas across different domains, such as Mountain Provinces in the Mediterranean Division (M260) and Arizona-New Mexico Mountains Semidesert-Open Woodland—Coniferous Forest—Alpine Meadow Province (M313)—appear in the same narrow distribution and fast convergence group (Fig. 7). At the same time mountain forests of Oregon and Washington (M240, Marine Division Mountain Provinces), located in the western part of the Humid Temperature Domain, display a distinctive pattern of wide distribution and slow convergence. The low number of available remeasured plots (less than 150) in northern provinces within the Dry Domain did not allow us to conclusively employ our methodology. In particular, the Temperate Steppe Division (330) as well as its mountain provinces (M330) were not classified using the spectral gap criterion (Fig. 7). Forests in the southern part of the Dry Domain (Fig. 7), such as Tropical Subtropical Steppe and Desert Divisions (310, 320), are classified as not being driven by shade tolerance, consistently with their low biomass, low diversity and relatively old stands completely dominated by shade intolerant species (Fig. 5).

Qualitatively, the regions proposed to be primarily shade tolerance driven exhibit also transition matrices with high values concentrated primarily on the main diagonal, and secondarily on the upper diagonal above it (Fig. 6). In addition, considering only stands older than 20 years did not result in substantial differences in transition matrices or shade tolerance index distributions (Figure 3 in S5 Appendix), indicating that the classification obtained are robust and valid for older stands. We also compared our conceptual model with the dynamics of shade tolerance index using the chronosequence approach (Figure 1 in S5 Appendix). The obtained regressions complement our classification by demonstrating that ecoregions with narrow distributions do not display the expected decrease in the early stages followed by an increase during the understory reinitiation (Fig. 2). These regressions also demonstrate that the differences observed between ecoregions are not purely confounded by stand age, but that they result from different dynamics of shade tolerance.

## Discussion

The shade tolerance index introduced in this paper is designed as a quantitative measure of forest succession according to the classic theory based on gap dynamics and replacement of shade intolerant by shade tolerant species. This study shows that this index can be utilized to understand the forest stand dynamics in ecoregions where the classic theory is validated, as it represents forest succession scale. In particular, our results demonstrate that this index is in agreement with the continuum index developed by Curtis and McIntosh [24] as well as with gap model simulation [62]. However, there are several challenges in the interpretation of the shade tolerance index results, which are related to existence of different successional pathways, disturbance regime complexity, spatial heterogeneity and non-stationarity of environmental variables.

The shade tolerance axis computed for southern Wisconsin data is similar to the continuum index axis developed by Curtis and McIntosh [24]. In the work cited the authors have derived the continuum index and the climax adaptation numbers based on extensive empirical observations of forest succession patterns in southern Wisconsin and measurements of species relative abundance within the stand without using the concepts of shade tolerance and gap dynamics. The shade tolerance index is based on the mechanistic succession model, as opposed to an empirically defined continuum index. Several attempts to redefine the continuum index following [24] in other ecoregions have produced mixed results, unsuccessful [72] and successful [52]. The major problem when developing a continuum index is the existence of several alternative successional pathways which coexist within the same mosaic of forest stands [72, 73]. Indeed, Curtis and McIntosh [24] considered only one successional pathway to create their continuum index and they were successful, as this was the primary successional pathway in southern Wisconsin [72]. Similarly, Nakamura [52] has successfully applied similar technique to study the single *Larix-Abies-Tsuga* successional pathway on the Mount Fuji forest. However, Kessell and Potter [73] demonstrated the existence of two distinct successional pathways that branch from the same conditions, and obviously the continuum index cannot be constructed in this case. Overall, the existence of several successional pathways is considered as a major challenge for modeling of forest succession [5, 74–77]. The advantage of the shade tolerance index is that it is based on a mechanism operating independently from the species composition of forest plots. The index can be used to describe the successional patterns in ecoregions where several alternative species replacement pathways coexist. In this case, a direct inversion of the shade tolerance index is not possible as different plots may have the same shade tolerance index level and very different species composition.

Our study of the FIA database led to the observation of highly similar correlation patterns across time and space (Figures 3–6 in S4 Appendix). It is worth emphasizing that (a) the correlation structure is similar across different ecoregions, and that (b) it is not affected by the change in the forest sampling protocol that was adopted nationwide in 1999. It is further highly remarkable that the correlation structure is similar to the one obtained in Eastern Canada in a previous work [69], despite substantial differences between the two forest inventories (e.g. climatic variables, species compositions, permanent plot designs, sampling protocols, experimental method used to determine the age of trees, stand biomass calculation). Overall, the preserved correlation patterns point toward important structural similarities between forested ecosystems in most of North America, and support the view that the shade tolerance index is a meaningful dimension that completes the usual indicators of biomass, basal area, implied stand age, and biodiversity.

We study the dynamics of the shade tolerance index in a Markov chain framework applied to the forest stand mosaic [11, 69]. Our approach is however fundamentally different from prior works using Markov chains to model succession as a change of species composition. For example, traditional Markov chain models [78] are based on expert knowledge of species transitions; empirically-based models [79, 80] are deduced from a substantial collection of species observations under the assumption that the Markov chain is stationary; mechanistically-based models [1, 81] predict overstory changes using a detailed survey of the understory; longer time scale Markov chains predicting forest types transition were developed in [82, 83]. In contrast with all these models, we consider the shade tolerance index instead of the specific species composition, as the former measure describes a forest stand in a more generic way than the latter. In particular, this approach allows the comparison of succession in ecoregions with highly different species composition.

Our analysis indicates that the classic succession theory is not universally valid for all ecoregions (Fig. 7). This is in agreement with other studies, in particular Chen and Taylor [84] recently demonstrated that simple assumptions about shade tolerance succession are not verified in Canadian boreal forests. Our analysis reveals that shade tolerance driven succession is linked with climatic and landscape factors that determine the division level classification of ecoregions [68, 71]. The most evident result concerns the eastern part of the Humid Temperate Domain, where we report a North—South monotonic transition from shade tolerance driven succession to other types of forest succession. This transition pattern is even more explicit if we exclude from consideration mountain areas, where the patch mosaic analysis should be modified to include altitude related climatic variables (Figure 2 in S4 Appendix). We hypothesize that the observed transition is caused by the climatic transition to more arid conditions [68]. This is partially supported by the fact that shade tolerance ranking is negatively correlated with the drought tolerance ranking [26]. Another supporting fact is that southernmost forests in the Dry domain are completely dominated by shade intolerant species. Our classification method is data dependent and we were able to investigate provinces with more than 150 resampled plots available. This restriction did not allow us to make a definite statement about shade tolerance driven succession in the western part of the Humid Temperate Domain and Nothern part of the Dry Domain where mountain areas are extended. However, the classification based on only one criterion, the width of the shade tolerance distribution (Fig. 7), indicates that shade tolerance may play an important role in these ecosystems. Overall, our analysis demonstrates that shade tolerance succession can be quantified at the landscape scale and connected with climatic variables. Alternatively, patterns in shade tolerance index might be connected to difference in forest management practices. In particular, intensive forest exploitation in southeastern US likely could result in younger stand age distributions at the landscape level, which in turns would result in a narrower shade tolerance distribution width. However, the data demonstrate that the correlation between stand age and shade tolerance index is overall weak (95% confidence interval [0.11, 0.13], c.f. Figure 3 in S4 Appendix) for all ecoregions (Figure 4 in S4 Appendix). The two largest southeastern ecoregions “231” and “232” are not exceptions to this rule, and also display weak correlations between stand age and shade tolerance (with respective 95% confidence intervals [0.11, 0.12] and [0.17, 0.19]). These weak correlations suggest that small differences in stand age should not have a big impact on shade tolerance distribution widths; however the nature of the observed differences in shade tolerance patterns between southeast and northeast US definitely deserves further investigation. We also anticipate that our approach can be extended to other successional mechanisms.

The gap dynamics and replacement of shade intolerant species by shade tolerant species is the essential successional mechanism employed in forest gap models such as JABOWA-FORET, SPACE, ZELIG and SORTIE models, their modifications and alternatives [6, 46, 62–65, 85–88]. These models simulate forest succession using the growth/mortality trade-off between fast growing shade intolerant early successional species and slower growing shade tolerant species as a mechanism of tree replacement and canopy recruitment [38, 46, 89]. In particular, the computer model that we used [62] follows idealized succession dynamics that rely mostly on gap-filling mechanisms and light-driven competition. Scaling methods approximating forest gap dynamics also employs the shade tolerance driven forest dynamics as the primary successional mechanism. Kohyama [90, 91] has employed the patch-mosaic concept for scaling forest gap dynamics to the landscape level. In the Kohyama’s model the forest succession is driven by the simple birth-and-disaster process presented as the conservation equation proposed by Levin and Paine [92]. The current study may call for substantial modifications of individual based models and for their validation in ecoregions where shade tolerance is not the main mechanism of succession.

In the ecoregions where the classic successional model is valid, the shade tolerance index naturally connects individual trees and stand succession. This provides an opportunity to understand forest dynamics across forest hierarchical structure [11, 88] using the hierarchical patch-dynamics concept [10]. In this framework, vegetation dynamics result from interactions of processes occurring on several hierarchical scales (or levels) including individual trees, forest stands and landscape units driven by natural and anthropogenic disturbances of different magnitudes. Individual-level dynamics within the stands is described using the individual-based models or their approximations [62], and the forest stand models are based on the patch-dynamics equations [11, 92].

We relied on the FIA database extensively throughout our analyses. In this study we operate at a large scale, with the aim to introduce the shade tolerance index as a generally meaningful macroscopic characteristic of forest plots. In order to include all ecological complexity and variations recorded in the FIA dataset, and avoid possible bias associated with filtering, our analysis included both managed and unmanaged forests. Despite this, we observed a good agreement with the study of Curtis of McIntosh in which plots were carefully selected [24], and with a previously published individual-based model [62]. In addition, using a large number of plots reduces the overall uncertainty associated with variations in individual plots. However additional filtering of the data restricted to specific classes of plots (e.g. ownership or land-use practices) can potentially improve the predictive power of the shade tolerance index.

## Supporting Information

S1 Appendix Statistical Analysis of Forest Inventory Data. Computation of the Shade Tolerance Index
(PDF)

S2 Appendix Analysis of Forest Succession in southern Wisconsin. (PDF)

S3 Appendix Succession in White Pine—Eastern Hemlock forests. (PDF)

S4 Appendix Correlation Analysis of Forest Stand Characteristics across the US ecoregions. (PDF)

S5 Appendix Supplementary Results for the classification of the US Ecoregions. (PDF)

S6 Appendix Shade Tolerance Rankings for Tree Species in the FIA Database. (CSV)
