# Supplementary material for: An Appraisal of the Classic Forest Succession Paradigm with the Shade Tolerance Index
Source: PLoS One. 2015 Feb 6;10(2):e0117138. doi: 10.1371/journal.pone.0117138 (PMC4319751; doi:10.1371/journal.pone.0117138)
Supplement: S1 Appendix — Computation of the Shade Tolerance Index (PDF) [file pone.0117138.s001.pdf]

APPENDIX 1

Statistical Analysis of Forest Inventory Data and Computation of the Shade Tolerance Index

Supplement to the article "*An appraisal of the classic forest succession paradigm with the shade-tolerance index.*"

Jean Lienard [1], Ionut Florescu[2], Nikolay Strigul[1*],

[1]- Department of Mathematics & School of Art and Sciences, Washington State University Vancouver.
[2]- Financial Engineering Division and the Hanlon Financial Systems Lab, Stevens Institute of Technology, Hoboken, NJ, USA
[*]- nick.strigul@wsu.edu

1 Statistical analyzes of the FIA database

The Forest Inventory and Analysis (FIA) is a forest survey program of the US department of Agriculture (Forest Inventory and Analysis Program, 2010). In this study employs a freely available FIA database that was downloaded from: `http://www.fia.fs.fed.us/`. Standard stand-level characteristics (biomass, stand age, and basal area) were computed according to the FIA manual (Forest Inventory and Analysis Program, 2010), other commonly used forest characteristics (Gini-Simpson and Shannon diversities) were computed according the procedures described elsewhere (Strigul et al., 2012, Lienard et al., 2014), and the computation of the original shade tolerance index is described in the next section. In particular, we extracted the following information about the trees of each plot, from Table `TREE`:

- The status of each tree (`STATUSCD`). We analyzed only trees with this variable set to 1 as we consider only live trees in this study.
- The carbon estimate above ground (`CARBON_AG`) and below ground (`CARBON_BG`). These variables are used to deduce the biomass as: 2 × (`CARBON_AG` + `CARBON_BG`).
- The diameter at breast height (`DIA`), used to compute the basal area as: $\pi \times$ `DIA` ^{2}.
- The tree species (`SPCD`), used to link with the shade tolerance ranking table, provided as a supplemental file.
- For the analysis of plot dynamics, we used the year of the survey (`INVYR`), as well as locations variables used in the computation of a unique identifier for each plot (`PLOT`, `COUNTYCD`, `UNITCD`, `STATECD`).
- The scaling factor (`TPA_UNADJ`), allowing to normalize plots of different sizes.

We also extracted information of plots from Table `COND`:

- The stand age (`STDAGE`), which is computed as the average of trees in a stand. We refer to this variable as the implied stand age in the text of the manuscript.
- The soil type (`PHYSCLCD`), used to select plots located on mesic soils.

In addition to these variables, we extracted from Table `PLOT` the following:

- Latitude (`LAT`) and longitude (`LON`) of plots.
- The ecoregion code (`ECOSUBCD`), from which we retained only the first four characters to derive Bailey's provinces (including a possible starting space, e.g., "M211" or " 211"). See Figure 1 in Appendix 4 for a map of the provinces as reported in the database.

The Gini-Simpson diversity index (Simpson, 1949) is equal to $1 - \sum_{s \in S} \left(\frac{\sharp(s)}{\sharp(T)} \right)^2$, with S being the set of species in a stand, $\sharp(s)$ being the number of trees with species s and $\sharp(T)$ being the total number of trees. The Shannon diversity index (Shannon and Weaver, 1949) is equal to $- \sum_{s \in S} \frac{\sharp(s)}{\sharp(T)} \ln \left(\frac{\sharp(s)}{\sharp(T)} \right)$, with the same notations. Both the Gini-Simpson and the Shannon indexes are in the 0-1 range, where high values indicate a high species heterogeneity.

Outliers in the database were removed according to the guidelines presented in Strigul et al. (2012).

2 Computation of the shade tolerance index using FIA data

We have explored several alternatives to calculate Ω_j, the abundance of tree species for a stand: 1) number of trees (canopy or understory) of the species j, 2) sum of the basal areas of the species j trees (total, canopy or understory), 3) biomass of the species j, 4) area of canopy occupied by species j. However, we discovered that these estimates are highly correlated (Appendix 4). Therefore is sufficient to compute only one of alternative Ω_j. In this study, we employed the abundance based on the sum of basal areas for the following reasons: a) the sum of basal areas is widely used in forestry for this purpose, b) statistically basal area is often related to crown area, and, therefore, the sum of basal areas can be also related to the canopy area occupied by species j (which is not recorded in the FIA dataset), c) this parameter is easy to calculate from the FIA dataset.

All the tree species in the FIA database were classified according to the shade tolerance tables (Baker, 1949, Burns and Honkala, 1990, Humbert et al., 2007) in 5 categories with respect to the shade tolerance: very intolerant, intolerant, intermediate, tolerant, and very tolerant. We assume that the scale of shade tolerance is linear and the classification of shade tolerance uniformly partition this scale, similarly to Humbert et al. (2007), Kunstler et al. (2009), Valladares and Niinemets (2008). Then we assign the following numbers to the shade tolerance classes: very intolerant = 0, intolerant = 0.25, intermediate = 0.5, tolerant = 0.75, and very tolerant = 1. Notice that this scale is opposite to the one of Humbert et al. (2007), which took 0 for the most tolerant and 1 for the most intolerant.

The shade tolerance rankings for North American tree species along with the species code from the FIA database is provided in a comma-separated file (online Appendix 6).

3 Statistical description of US forests across ecoregions

Table 1: Shade tolerance index distribution across US provinces.

Province	Min.	1st Qu.	Median	Mean	3rd Qu.	Max.
211	0	0.525	0.6794	0.6615	0.8273	1
212	0	0.261	0.4882	0.491	0.7258	1
221	0	0.3913	0.4982	0.4985	0.6006	1
222	0	0.3458	0.4863	0.4691	0.5908	1
223	0	0.3316	0.4349	0.4311	0.5196	1
231	0	0.2552	0.327	0.3496	0.4282	1
232	0	0.2795	0.3887	0.3971	0.5	1
234	0	0.3203	0.4393	0.4297	0.527	1
242	0	0.4829	0.5138	0.5534	0.6491	1
251	0	0.3498	0.4603	0.4538	0.5378	1
255	0	0.25	0.3306	0.3347	0.4569	1
261	0.07565	0.5	0.5876	0.6367	0.8038	1
262	0.119	0.1331	0.25	0.3391	0.5	0.6936
263	0.09627	0.5993	0.7001	0.7028	0.8333	1
313	0	0.007809	0.0791	0.1314	0.2243	0.75
315	0	0	0	0.1114	0.1719	1
321	0	0	0	0.07703	0.01074	1
322	0	0	0	0.04668	0.02958	0.7153
331	0	0.06614	0.25	0.2444	0.2776	1
332	0	0.1287	0.378	0.3534	0.5424	1
341	0	0	0.008568	0.09427	0.118	1
342	0	0.003473	0.25	0.2192	0.25	1
411	0	0.5	0.5	0.5331	0.5958	0.75
M211	0	0.6618	0.7921	0.7592	0.8878	1
M221	0	0.3925	0.4833	0.4824	0.562	1
M223	0	0.2758	0.3915	0.3745	0.4763	0.8987
M231	0	0.1785	0.2636	0.2782	0.3617	1
M242	0	0.4515	0.5483	0.5623	0.7344	1
M261	0	0.2728	0.4666	0.445	0.5852	1
M262	0	0.146	0.2983	0.3248	0.5	0.75
M313	0	0.043	0.2029	0.1986	0.2825	0.9106
M331	0	0.1087	0.3447	0.355	0.5503	1
M332	0	0.2504	0.4414	0.405	0.5223	0.9818
M333	0	0.3139	0.4959	0.4826	0.6834	1
M334	0	0.25	0.25	0.2625	0.25	0.6526
M341	0	0	0.1007	0.1831	0.313	1

Table 2: Summary statistics of Biomass (in 10^3kg/ha) for each US province.

Province	Min.	1st Qu.	Median	Mean	3rd Qu.	Max.
211	0.09628	60.68	111.2	119.3	166.6	528.7
212	0.03543	41.21	76.53	84.58	119	542.5
221	0.08031	73.54	125.9	131.1	178.7	526.6
222	0.05631	39.66	80.04	90.39	127.4	521.1
223	0.08261	58.56	96.3	101.7	135.9	566
231	0.04592	49.93	97.01	105.9	149.7	557.5
232	0.07411	35.8	80.13	97.69	141.2	808.9
234	0.1585	45.57	97.58	116	165.8	870.3
242	0.08631	54.75	144.1	192.2	279.4	973.7
251	0.05393	39.06	73.08	81.18	114.1	578.3
255	0.1218	17.97	43.14	54.51	79.36	308.8
261	0.2588	40.05	125.7	216.6	324.5	1271
262	11	27.37	80.69	84.18	144.3	157.5
263	1.024	125.2	227.4	275.1	360.1	1787
313	0.1743	11.6	24.82	37.13	46.89	464.2
315	0.08919	5.123	11.46	18.71	25.05	191.5
321	0.08249	2.154	5.369	11.19	11.96	360.8
322	0.1633	6.199	13.32	20.59	25.14	133.5
331	0.04699	10.98	25.6	37.13	50.39	302.1
332	0.1892	24.7	48.04	61.57	83.42	608.1
341	0.08775	12.17	24.78	32.78	42.84	337.5
342	0.08842	7.497	16.55	30.76	37.79	377.9
411	0.1656	21.61	56.92	72.16	102.9	312.6
M211	0.04047	72.11	121.2	125.3	171.2	457.7
M221	0.08035	94.63	141.8	146.8	192.5	588.2
M223	0.5212	70.45	107.9	108.9	146.9	378.1
M231	0.1051	47.92	84.5	90.49	125.8	329.1
M242	0.09882	71.22	178.2	247.1	363.4	1651
M261	0.07831	49.36	125.9	174.1	250.2	1287
M262	0.205	16.16	44.43	65.55	98.48	355.7
M313	0.1894	14.17	34.04	51.58	69.75	368.6
M331	0.1049	29.12	62.85	80.99	115.3	500.3
M332	0.08631	37.21	78.85	96.64	133.5	650.1
M333	0.1137	45.97	99.03	121.6	168.8	670.7
M334	0.1024	31.52	57.61	66.83	91.94	275.3
M341	0.1848	16.04	30.98	41.49	54.03	413.3

Table 3: Summary statistics of Basal area (in m^2/ha) for each US province.

Province	Min.	1st Qu.	Median	Mean	3rd Qu.	Max.
211	0.07509	14.47	22.96	22.67	30.36	75.17
212	0.03755	11.75	19.37	19.84	26.98	101.9
221	0.07509	13.19	20.45	20.28	26.73	66.74
222	0.03755	9.036	16.36	16.87	23.57	67.26
223	0.04544	12.09	18.19	17.88	23.46	71.03
231	0.07504	11.93	19.74	19.52	26.54	80.53
232	0.07488	9.098	17.36	18.87	26.42	141
234	0.09386	10.15	18.39	19.51	26.71	130.7
242	0.09386	11.38	23.71	26.94	38.09	105.8
251	0.03755	8.609	15.01	15.53	21.43	86.31
255	0.09386	5.324	11.27	12.35	18.02	57.32
261	0.1136	9.35	20.13	31.18	45.23	138.3
262	1.089	5.561	9.014	16.4	30.75	35.58
263	0.5876	23.87	39.8	42.59	56.86	154.5
313	0.09386	8.185	15.13	17.64	24.69	79.7
315	0.09386	2.876	5.97	8.238	11.52	59.87
321	0.09386	1.736	3.961	6.371	8.279	70.69
322	0.3184	4.724	9.115	12.54	16.43	70.77
331	0.09386	5.157	10.33	12.54	17.77	90.88
332	0.1089	6.418	12.36	14.09	19.75	95.97
341	0.09386	9.174	16.84	19.16	26.65	83.45
342	0.09386	4.411	8.852	12.41	16.7	98.66
411	0.1884	6.892	16.27	19.38	27.69	66.15
M211	0.07509	17.11	24.83	24.47	31.81	64.18
M221	0.07513	17.46	23.66	23.47	29.6	68.54
M223	0.2713	14.66	19.22	19.12	24.36	47.45
M231	0.09386	12.12	18.24	18.24	24.3	49.02
M242	0.1136	16.96	31.7	35.26	49.75	139.1
M261	0.1136	12.66	25.88	29.55	42.74	136.4
M262	0.3692	5.42	10.56	14.76	21.74	62.99
M313	0.2112	9.843	17.68	19.05	26.07	76.69
M331	0.1136	11.54	20.18	22.27	31.17	104.8
M332	0.09386	10.95	19.48	21.23	29.29	108.1
M333	0.1136	11.87	21.73	23.71	32.54	106.6
M334	0.2117	9.553	16.11	16.83	22.45	49.31
M341	0.1136	10.21	18.69	20.6	28.45	92.67

Table 4: Summary statistics of Average age of trees (implied stand age) for each US province.

Province	Min.	1st Qu.	Median	Mean	3rd Qu.	Max.
211	1	45	65	62.62	82	195
212	1	35	55	54.99	73	272
221	1	43	59	57.54	73	203
222	1	35	51	54.18	70	311
223	1	40	56	56.16	71	162
231	1	16	35	37.65	55	195
232	1	15	33	38.87	55	195
234	1	30	50	46.92	64	170
242	1	21	45	49.64	70	350
251	1	35	50	52.35	68	206
255	1	25	40	41.85	55	130
261	2	74.75	90	113.4	126.2	600
262	50	60	60	70	80	100
263	1	40	55	67.6	80	500
313	2	93	134	141.9	184.5	522
315	1	25	40	45.04	55	550
321	1	25	45	66.68	92	318
322	5	77	110	121.9	160	390
331	1	50	74	75.7	95	328
332	3	35	50	50.94	65	130
341	2	90	130.5	137.9	180	1028
342	4	61	83	98.1	120	380
411	1	30	50	55.46	82	139
M211	1	46	66	65.17	83	238
M221	1	45	64	61.91	80	200
M223	2	50	63	58.54	71	111
M231	1	27	49	46.32	65	115
M242	1	31	65	96.16	120	800
M261	1	62	89	106.3	125	997
M262	2	70	94	110.8	150	325
M313	1	80	103	109.7	134	343
M331	1	67	98	102.2	134	733
M332	2	70	98	104.8	133	494
M333	1	48	79	82.87	109	404
M334	1	67	85	84.21	100	278
M341	2	78.75	118.5	125.7	168	548

Table 5: Summary statistics of Shannon diversity index for each US province.

Province	Min.	1st Qu.	Median	Mean	3rd Qu.	Max.
211	0	0.8676	1.197	1.148	1.476	2.242
212	0	0.6315	0.9831	0.9409	1.297	2.3
221	0	1.029	1.38	1.311	1.666	2.571
222	0	0.6909	1.089	1.043	1.43	2.465
223	0	1.169	1.512	1.44	1.779	2.694
231	0	0.9968	1.411	1.315	1.725	2.689
232	0	0.5228	0.9988	0.9405	1.395	2.509
234	0	0.7053	1.176	1.094	1.508	2.35
242	0	0.2206	0.6525	0.6472	1.03	1.709
251	0	0.76	1.192	1.134	1.542	2.657
255	0	0.4698	0.8777	0.8464	1.267	2.435
261	0	0	0.5943	0.5254	0.868	1.436
262	0	0	0.153	0.4195	0.8897	1.055
263	0	0.5656	0.8039	0.7863	1.048	1.648
313	0	0.1011	0.5236	0.4721	0.687	1.672
315	0	0	0	0.2756	0.5727	1.607
321	0	0	0	0.2322	0.4978	1.555
322	0	0	0.216	0.303	0.5985	1.446
331	0	0	0.1838	0.3134	0.6277	1.604
332	0	0.3386	0.6913	0.6951	1.053	1.822
341	0	0	0.4325	0.3832	0.6497	1.557
342	0	0	0	0.156	0.1753	1.375
411	0	0	0.4862	0.512	0.8577	1.891
M211	0	0.9022	1.214	1.175	1.49	2.272
M221	0	1.168	1.471	1.412	1.719	2.477
M223	0	1.251	1.547	1.486	1.77	2.421
M231	0	0.9651	1.338	1.263	1.621	2.356
M242	0	0.3185	0.6653	0.6375	0.9483	1.871
M261	0	0.2479	0.6667	0.636	0.9777	1.858
M262	0	0	0.3073	0.3629	0.6277	1.327
M313	0	0.2105	0.6365	0.5933	0.911	1.766
M331	0	0.08903	0.5353	0.4963	0.7655	1.714
M332	0	0.1124	0.5323	0.5088	0.7968	1.7
M333	0	0.3652	0.6894	0.691	1.015	1.871
M334	0	0	0	0.2283	0.4491	1.358
M341	0	0.1884	0.5598	0.4994	0.6906	1.596

Table 6: Summary statistics of Gini-Simpson diversity index for each US province.

Province	Min.	1st Qu.	Median	Mean	3rd Qu.	Max.
211	0	0.475	0.6209	0.5697	0.7181	0.8788
212	0	0.3529	0.5423	0.4893	0.668	0.8902
221	0	0.5384	0.6753	0.6208	0.762	0.9083
222	0	0.4069	0.5892	0.527	0.7068	0.9011
223	0	0.6036	0.72	0.6665	0.7901	0.9227
231	0	0.538	0.6941	0.6227	0.7799	0.9256
232	0	0.2981	0.5558	0.4815	0.7014	0.9075
234	0	0.415	0.6102	0.537	0.7252	0.8926
242	0	0.1072	0.4087	0.3635	0.5917	0.7844
251	0	0.4451	0.625	0.5588	0.7349	0.9202
255	0	0.2482	0.5	0.4466	0.6616	0.8967
261	0	0	0.3455	0.305	0.4999	0.7388
262	0	0	0.06827	0.2449	0.5161	0.64
263	0	0.3121	0.4952	0.4425	0.5998	0.791
313	0	0.03957	0.32	0.2908	0.48	0.7929
315	0	0	0	0.1671	0.3627	0.7812
321	0	0	0	0.1385	0.3013	0.7615
322	0	0	0.1049	0.1915	0.3912	0.7435
331	0	0	0.08318	0.1924	0.4086	0.7978
332	0	0.1725	0.4444	0.3872	0.595	0.8281
341	0	0	0.2512	0.2459	0.4444	0.78
342	0	0	0	0.09683	0.0811	0.7445
411	0	0	0.2864	0.2928	0.5089	0.8195
M211	0	0.4918	0.6289	0.5844	0.723	0.882
M221	0	0.5958	0.707	0.661	0.7768	0.905
M223	0	0.6358	0.7324	0.6912	0.7902	0.9031
M231	0	0.5297	0.6779	0.6156	0.758	0.8993
M242	0	0.1599	0.4182	0.3648	0.5468	0.8228
M261	0	0.1171	0.4061	0.3559	0.5582	0.8155
M262	0	0	0.1618	0.216	0.4102	0.6798
M313	0	0.0986	0.3969	0.3434	0.5301	0.8178
M331	0	0.0334	0.3171	0.2938	0.493	0.7902
M332	0	0.04519	0.3134	0.2981	0.4974	0.8047
M333	0	0.19	0.4322	0.3839	0.5714	0.8264
M334	0	0	0	0.1386	0.2604	0.7033
M341	0	0.08588	0.3476	0.3074	0.4851	0.7901

4 Spatial visualization of forest characteristics in decades

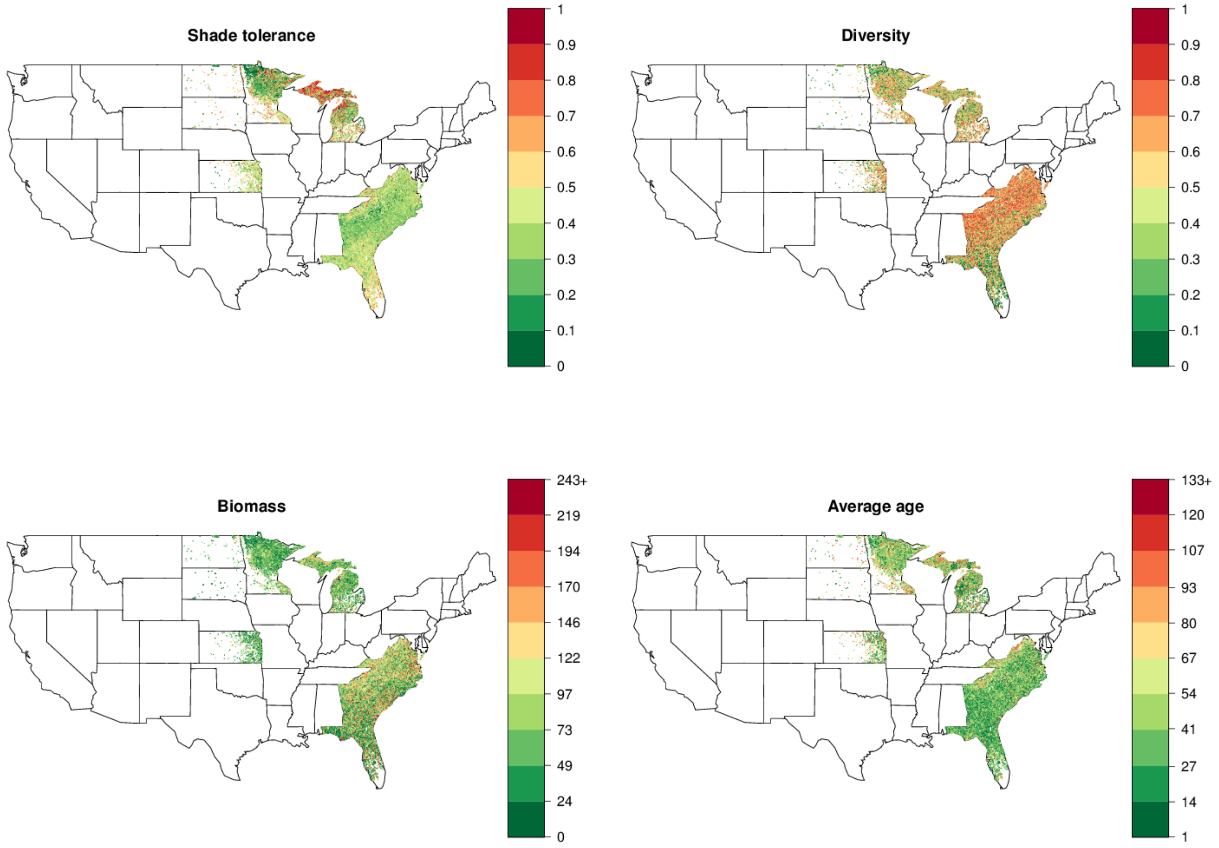

Figure 1: Stand-level characteristics of plots for 1968-1982.

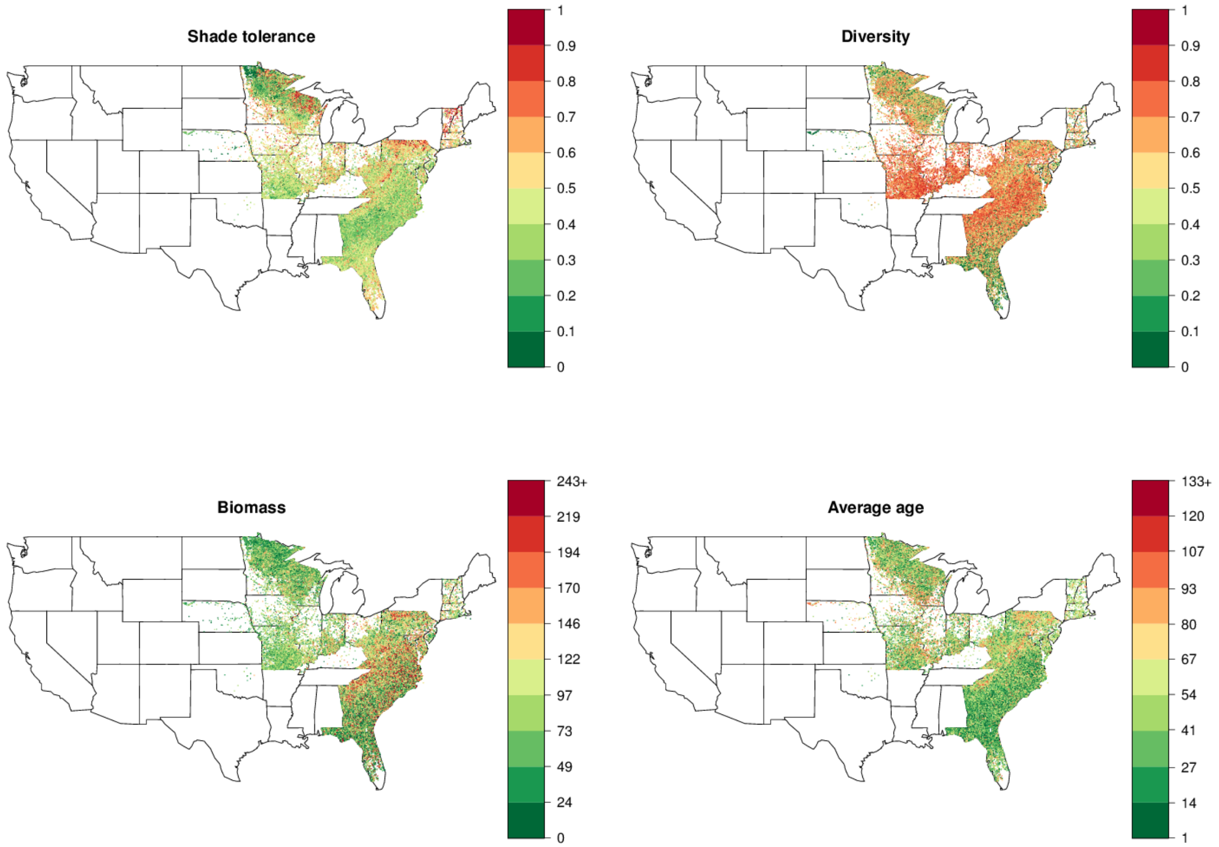

Figure 2: Stand-level characteristics of plots for 1982-1992.

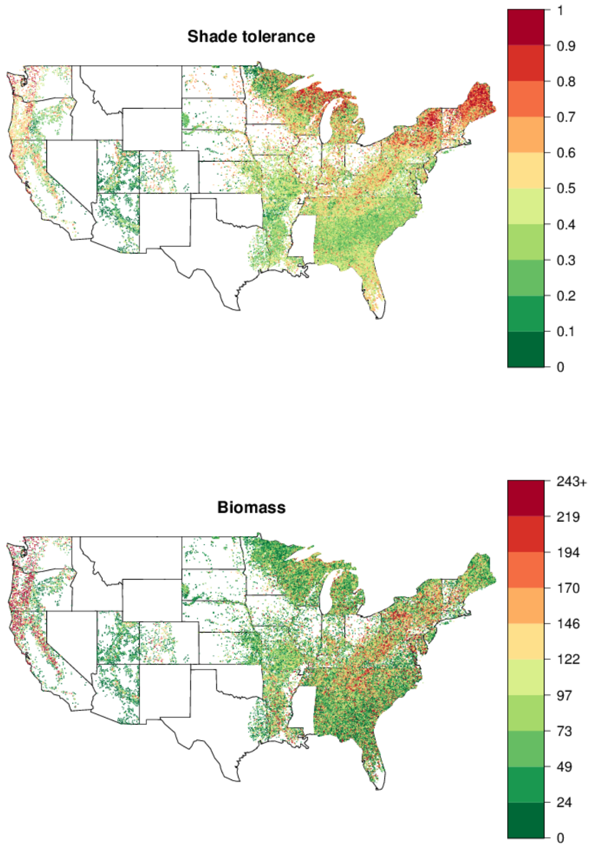

Figure 3: Stand-level characteristics of plots for 1992-2002.

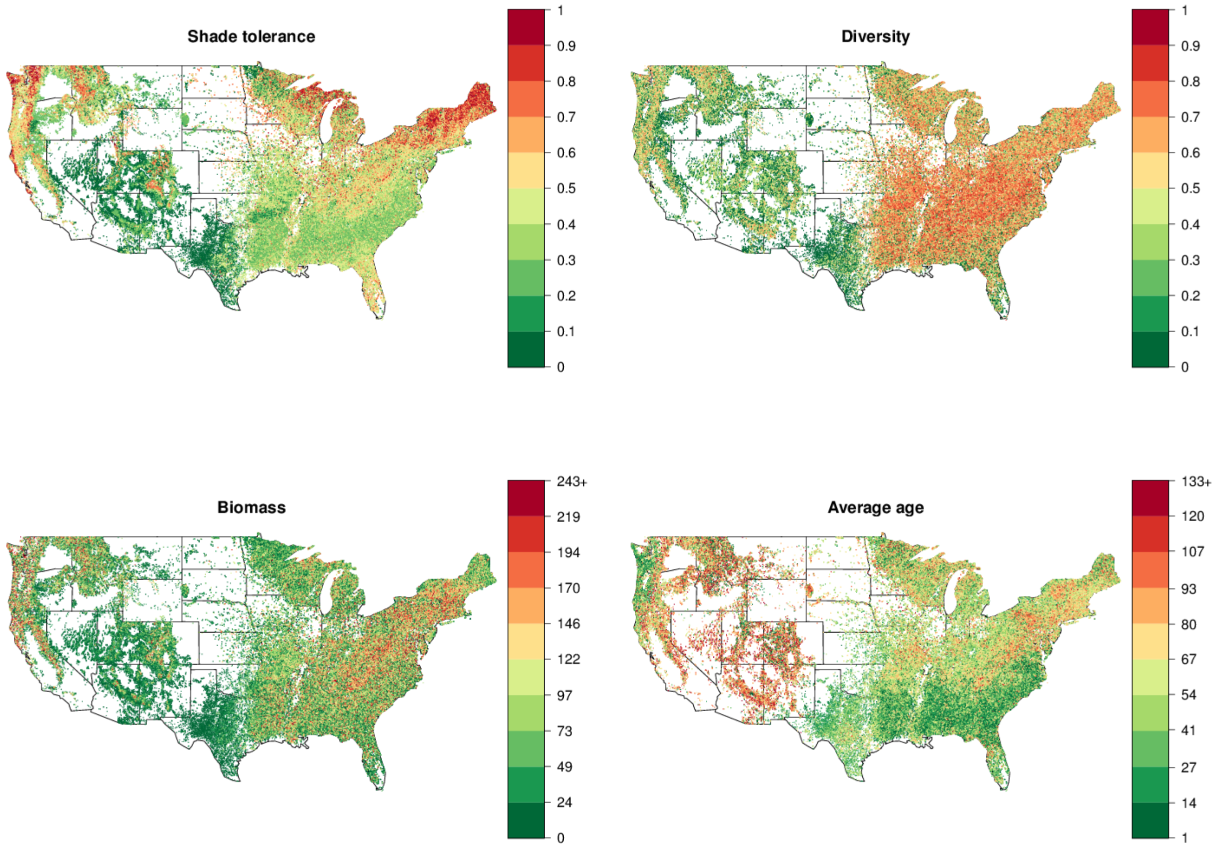

Figure 4: Stand-level characteristics of plots for 2002-2012.

References

Baker, F. S. (1949). A revised tolerance table. *Journal of Forestry*, 47(3):179–181.

Burns, R. M. and Honkala, B. H. (1990). *Silvics of North America. Volumes 1, 2.* US Dept. of Agriculture, Forest Service.

Forest Inventory and Analysis Program (2010). Database Description and Users Manual Version 4.0 for Phase 2 (Revision 3).

Humbert, L., Gagnon, D., Kneeshaw, D., and Messier, C. (2007). A shade tolerance index for common understory species of northeastern North America. *Ecological Indicators*, 7(1):195–207.

Kunstler, G., Coomes, D. A., and Canham, C. D. (2009). Size-dependence of growth and mortality influence the shade tolerance of trees in a lowland temperate rain forest. *Journal of ecology*, 97(4):685–695.

Lienard, J., Gravel, D., and Strigul, N. S. (2014). Data-intensive multidimensional modeling of forest dynamics. *http://dx.doi.org/10.1101/005009*.

Shannon, C. E. and Weaver, W. (1949). The mathematical theory of information.

Simpson, E. H. (1949). Measurement of diversity. *Nature*.

Strigul, N., Florescu, I., Welden, A. R., and Michalczewski, F. (2012). Modelling of forest stand dynamics using markov chains. *Environmental Modelling and Software*, 31:64 – 75.

Valladares, F. and Niinemets, Ü. (2008). Shade tolerance, a key plant feature of complex nature and consequences. *Annual Review of Ecology, Evolution, and Systematics*, 39(1):237–257.
