# Supplementary material for: An Appraisal of the Classic Forest Succession Paradigm with the Shade Tolerance Index
Source: PLoS One. 2015 Feb 6;10(2):e0117138. doi: 10.1371/journal.pone.0117138 (PMC4319751; doi:10.1371/journal.pone.0117138)
Supplement: S2 Appendix — (PDF) [file pone.0117138.s002.pdf]

APPENDIX 2

Analysis of Forest Succession in southern Wisconsin

Supplement to the article "*An appraisal of the classic forest succession paradigm with the shade-tolerance index.*"

Jean Lienard [1], Ionut Florescu[2], Nikolay Strigul[1*],

[1]- Department of Mathematics & School of Art and Sciences, Washington State University Vancouver.

[2]- Financial Engineering Division and the Hanlon Financial Systems Lab, Stevens Institute of Technology, Hoboken, NJ, USA

[*]- nick.strigul@wsu.edu

1 Continuum Index and Shade Tolerance Index in Wisconsin

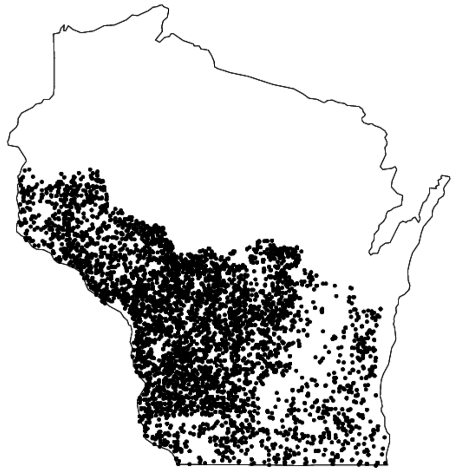

Figure 1: Plot locations in southern Wisconsin used for the comparison with Curtis and McIntosh (1951). The overall geographical area considered is similar to the Figure 1 in Curtis and McIntosh (1951), while the number of plots (n=7017) considered here is substantially larger than in the original study (n=95).

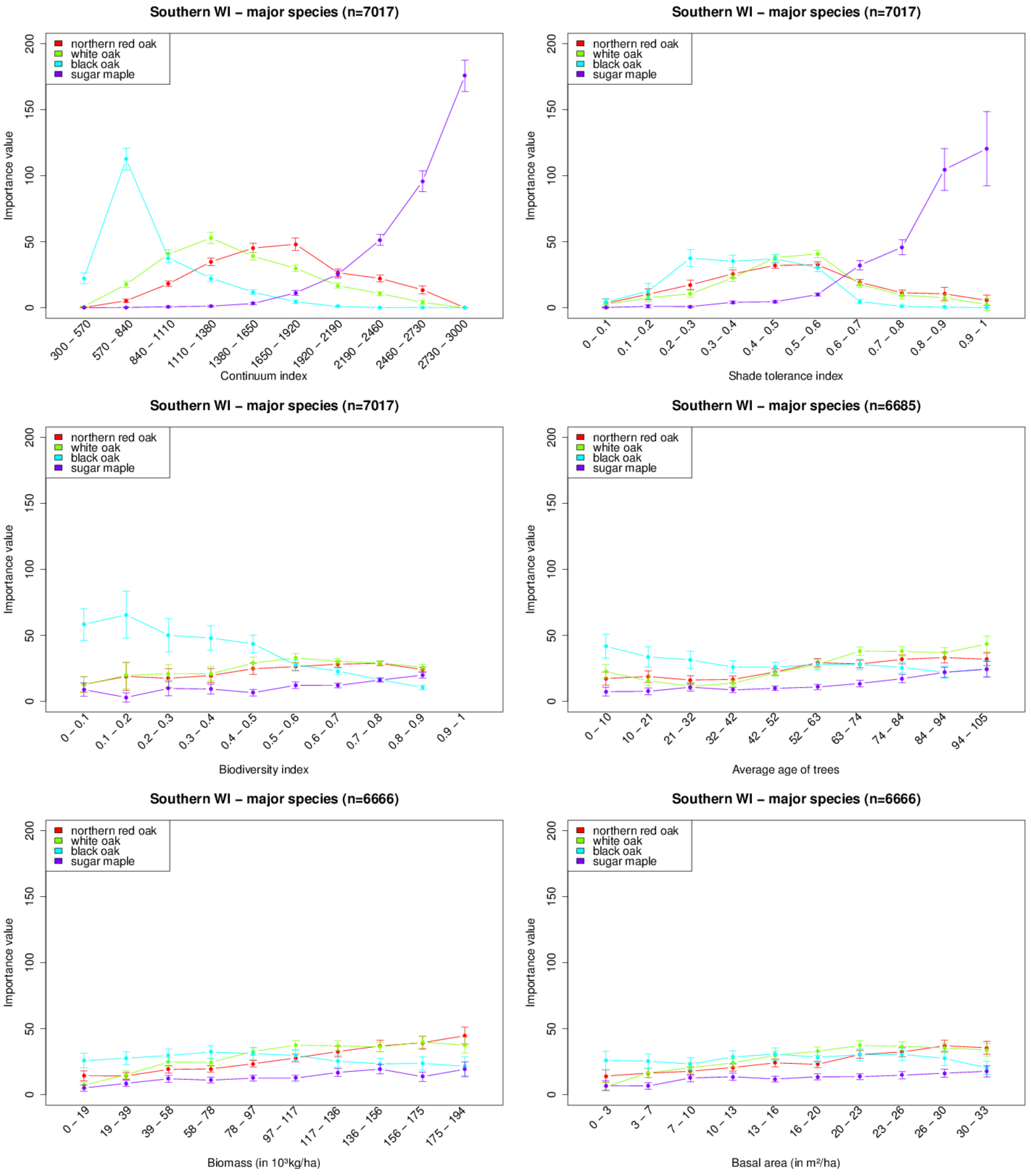

Figure 2: Characteristics for the major species in southern Wisconsin. Bars indicate the standard error of the mean.

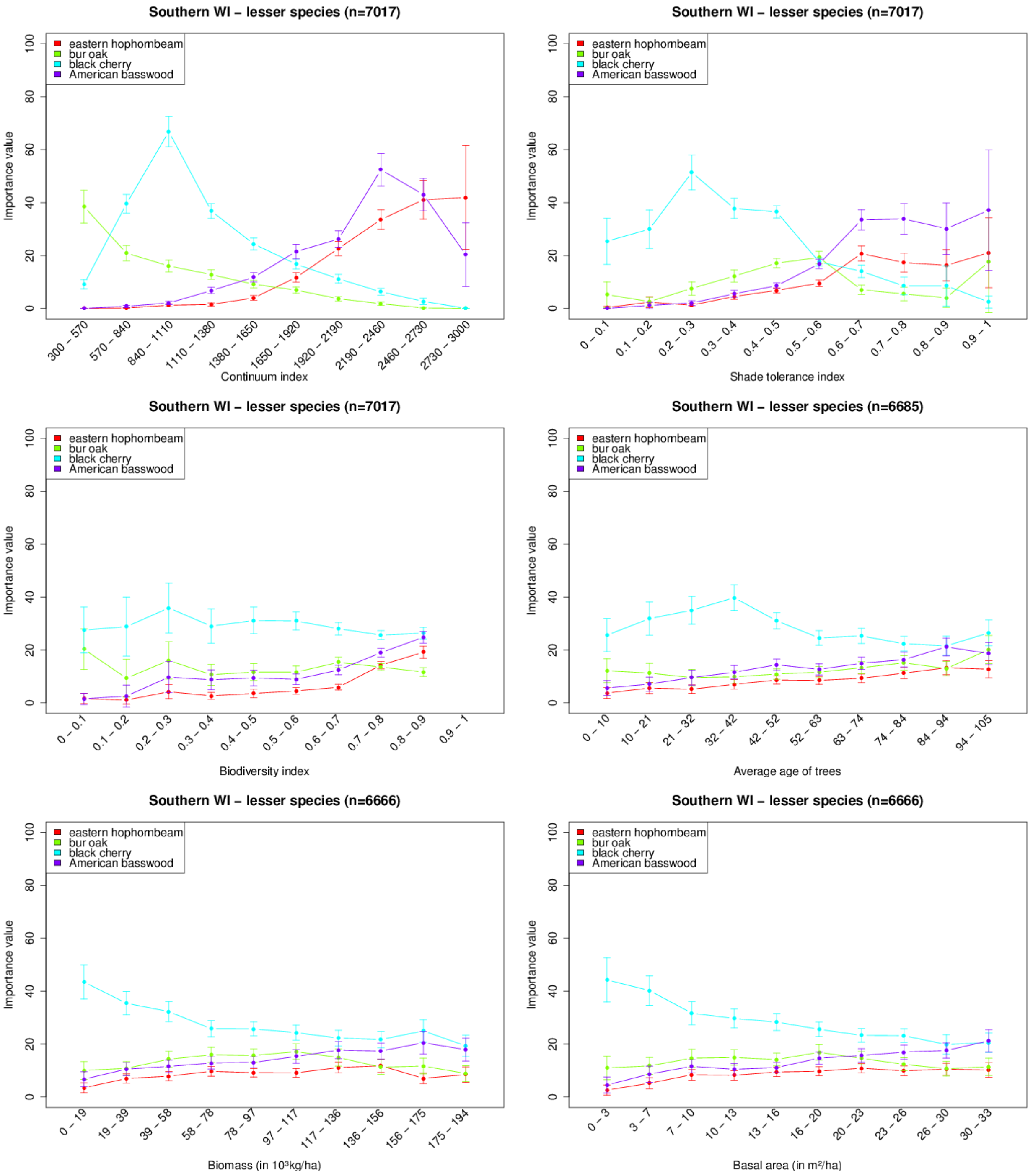

Figure 3: Characteristics for the lesser species in southern Wisconsin. Bars indicate the standard error of the mean.

2 Correlation analysis of the Continuum and Shade tolerance indices

The scatterplot of shade tolerance index and continuum index displays a relationship in all plots studied (Figure 4). In addition, breaking down each of the 7017 stands according to their dominant species highlight the correspondance between both indices (Figure 5).

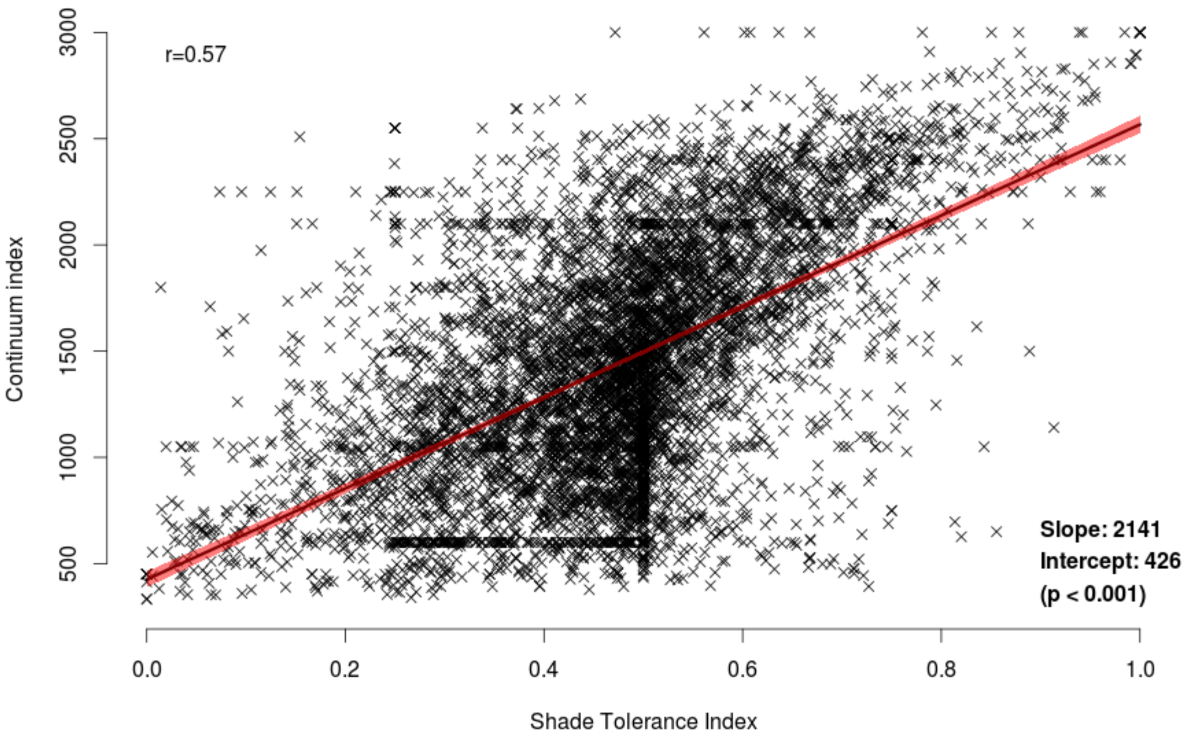

Figure 4: Scatterplot of the Shade Tolerance and Continuum indices in Southern Wisconsin for all 7017 plots studied. Both indices are correlated with a correlation coefficient of 0.57 within a 95% confidence interval $[0.55, 0.59]$.

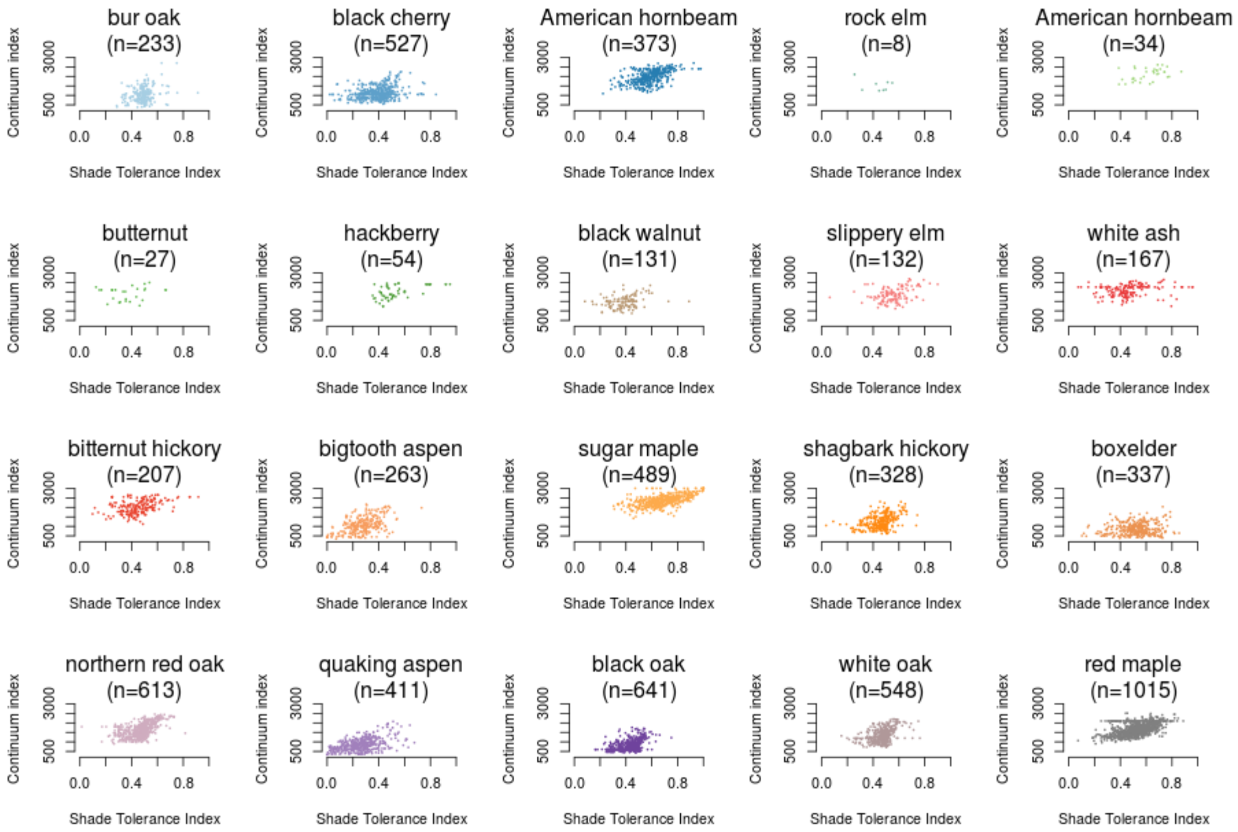

Figure 5: Scatterplot of the Shade Tolerance and Continuum indices in Southern Wisconsin, broken down according to the most represented species in each plot. The correlated patterns displayed here strengthen the overall correlation obtained using all plots (Figure 4).

References

Curtis, J. T. and McIntosh, R. P. (1951). An upland forest continuum in the prairie-forest border region of Wisconsin. *Ecology*, 32(3):476–496.
