# Supplementary material for: An Appraisal of the Classic Forest Succession Paradigm with the Shade Tolerance Index
Source: PLoS One. 2015 Feb 6;10(2):e0117138. doi: 10.1371/journal.pone.0117138 (PMC4319751; doi:10.1371/journal.pone.0117138)
Supplement: S3 Appendix — (PDF) [file pone.0117138.s003.pdf]

APPENDIX 3

Succession in White Pine – Eastern Hemlock forests

Supplement to the article "*An appraisal of the classic forest succession paradigm with the shade-tolerance index.*"

Jean Lienard [1], Ionut Florescu[2], Nikolay Strigul[1*],

[1]- Department of Mathematics & School of Art and Sciences, Washington State University Vancouver.
[2]- Financial Engineering Division and the Hanlon Financial Systems Lab, Stevens Institute of Technology, Hoboken, NJ, USA
[*]- nick.strigul@wsu.edu

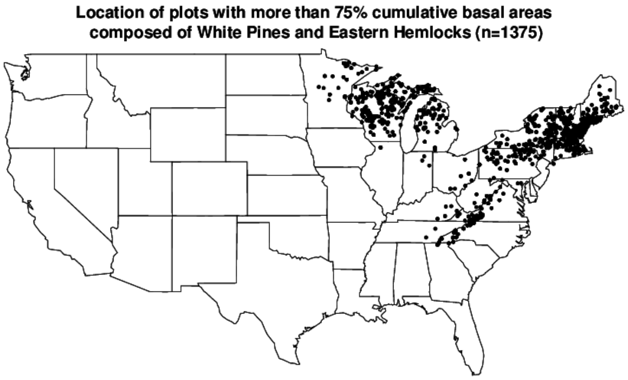

Figure 1: Map of the US showing the location of White Pine – Eastern Hemlock two-species systems (studied in the comparison with the computer model of Strigul et al. (2008)).

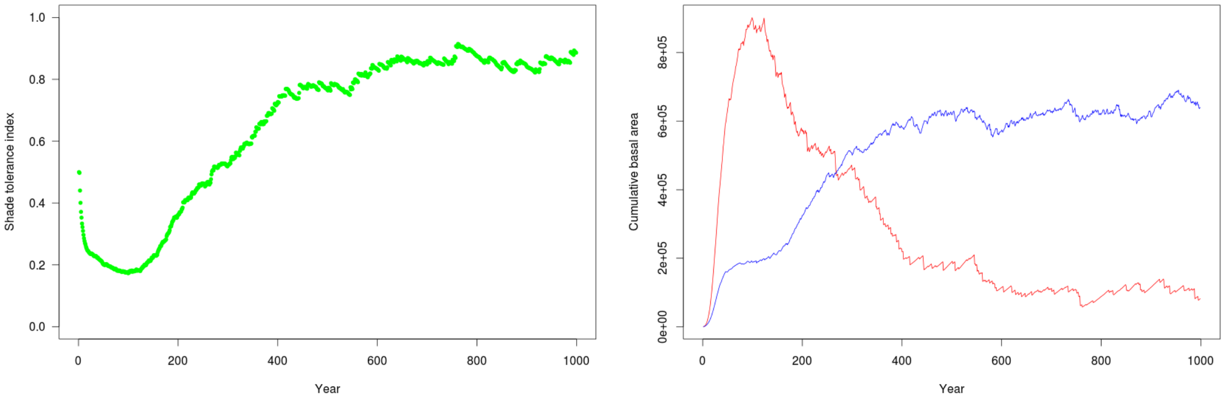

Figure 2: Computer simulation of one hectare containing White Pine – Eastern Hemlock forests (1 hectare, 1000 years), as in the figure shown in main text, but with the x-axis representing time after a major disturbance. Left: *shade tolerance index.* Right: *cumulative basal area of white pines (blue) and eastern hemlock (red).*

References

Strigul, N., Pristinski, D., Purves, D., Dushoff, J., and Pacala, S. (2008). Scaling from trees to forests: tractable macroscopic equations for forest dynamics. *Ecological Monographs*, 78(4):523–545.
