# Supplementary material for: An Appraisal of the Classic Forest Succession Paradigm with the Shade Tolerance Index
Source: PLoS One. 2015 Feb 6;10(2):e0117138. doi: 10.1371/journal.pone.0117138 (PMC4319751; doi:10.1371/journal.pone.0117138)
Supplement: S4 Appendix — (PDF) [file pone.0117138.s004.pdf]

APPENDIX 4

Correlation Analysis of Forest Stand Characteristics across the US ecoregions

Supplement to the article "*An appraisal of the classic forest succession paradigm with the shade-tolerance index.*"

Jean Lienard [1], Ionut Florescu[2], Nikolay Strigul[1*],

[1]- Department of Mathematics & School of Art and Sciences, Washington State University Vancouver.

[2]- Financial Engineering Division and the Hanlon Financial Systems Lab, Stevens Institute of Technology, Hoboken, NJ, USA

[*]- nick.strigul@wsu.edu

1 Bailey's provincial subdivision of the US

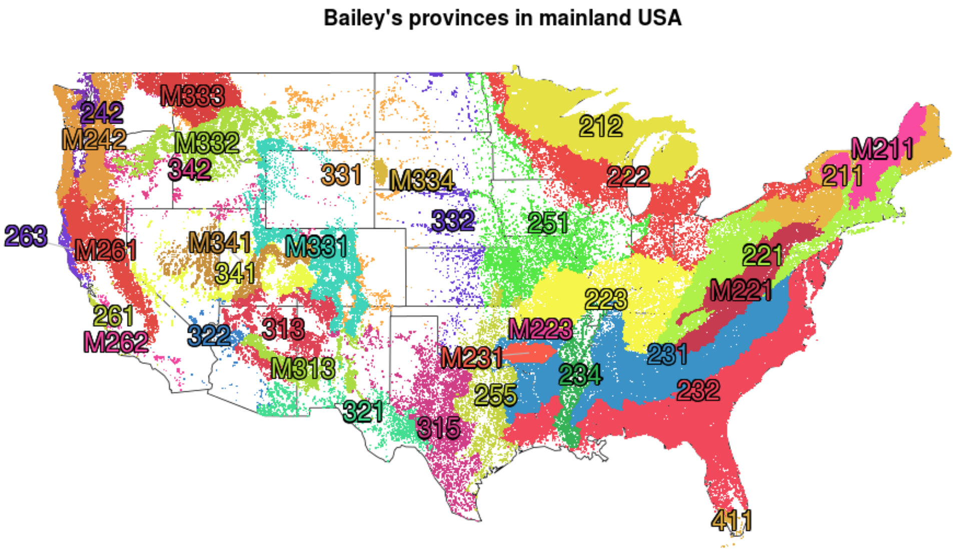

Figure 1: Bailey's provinces (Bailey, 1995), as reported in the FIA database (first four characters from column `ECOSUBCD` *of table* `PLOT`*). Each dot corresponds to one measured stand; different colors were randomly chosen to allow the distinction between provinces.*

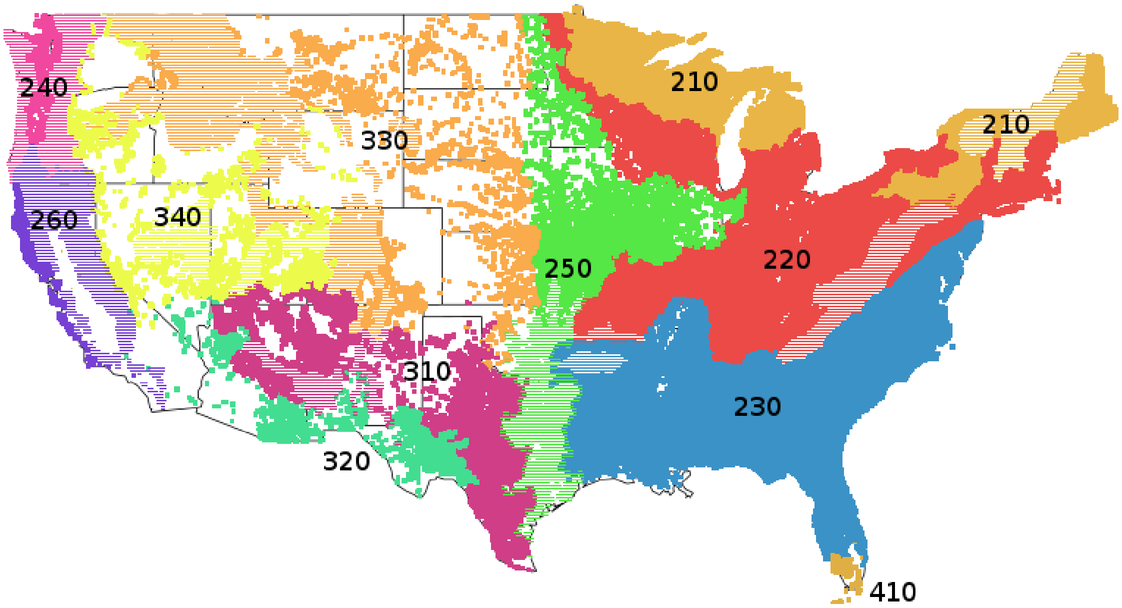

Figure 2: Ecoregion divisions of the US forested ecosystems (see also Bailey, 1995, and Figure 1 in this appendix). The first digit "2" indicates divisions inside the Humid Temperate Domain (e.g. 210), "3" inside the Dry Domain and "4" inside the Humid Tropical Domain. Dashed areas correspond to mountain provinces.

2 Overall correlations patterns of forest characteristics

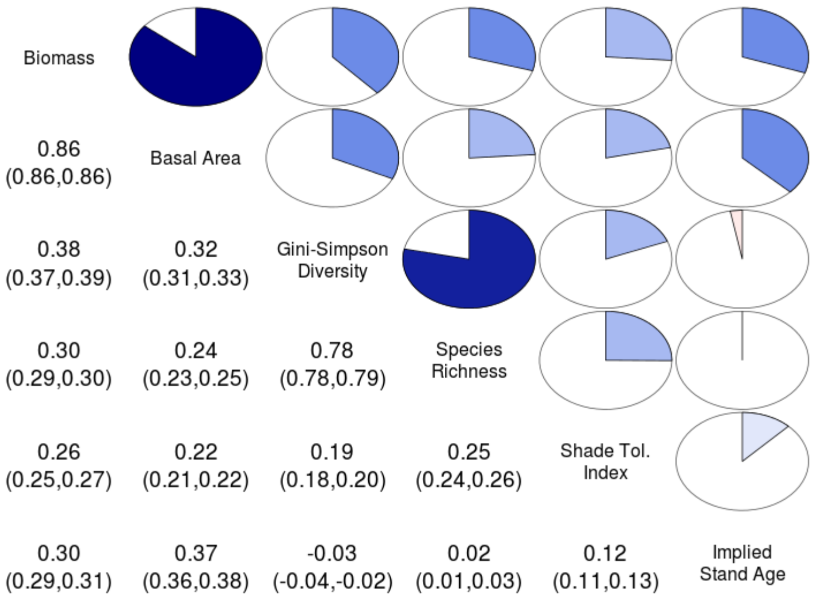

Figure 3: Stand characteristics correlation matrix based on the whole database; the numbers below the diagonal are the Pearsons coefficients (with 95% confidence intervals) and the circles above the diagonal provide visual indications of the correlations. Breakdowns for different ecoregions and different years are presented in following the Figures 6 and 5.

3 Correlations of different forest characteristics depending on computation methods

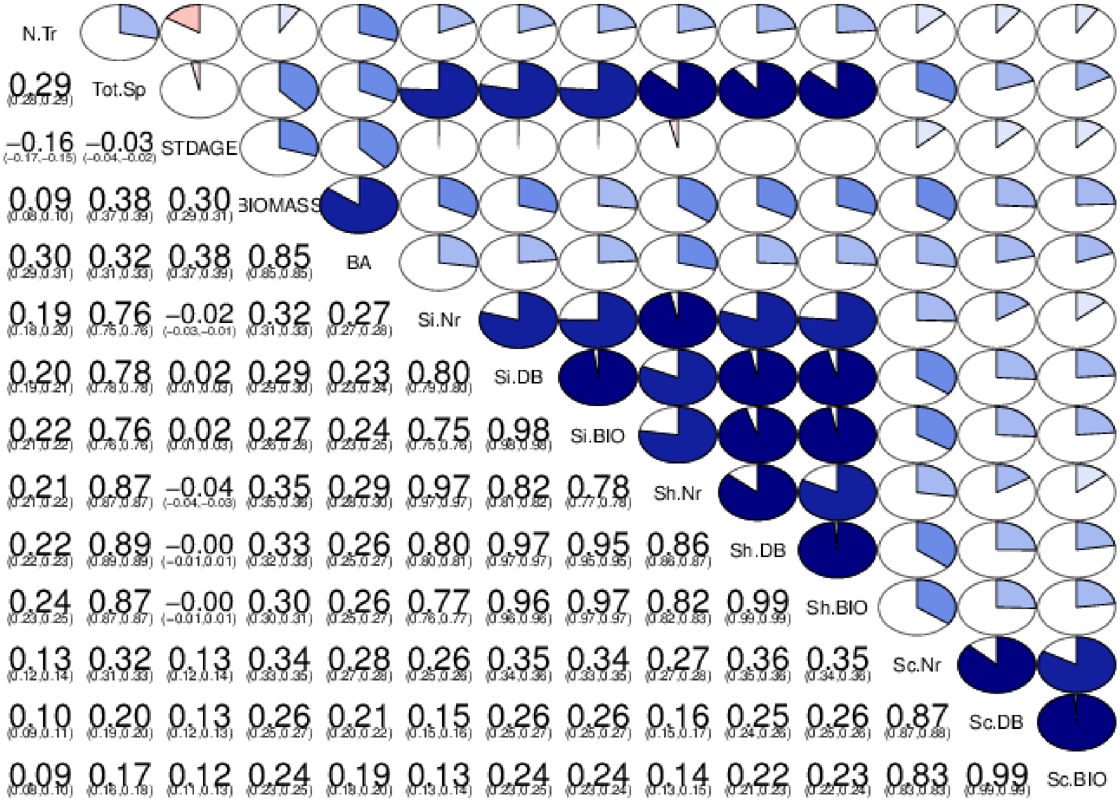

Figure 4: Correlations in the whole dataset using Pearson's coefficients represented graphically (upper diagonal) and numerically with 95% confidence interval (lower diagonal) (Friendly, 2002). Abbreviations: `N.Tr` is the number of trees per hectare; `Tot.Sp` is the species richness; `STDAGE` is the stand age as reported in the FIA database; `BIOMASS` is the total biomass per hectare; `BA` is the Basal Area per hectare; `Si.Nr`, `Si.DB` and `Si.BIO` are the Gini-Simpson diversity indexes based on number of trees, basal area and biomass respectively; similarly, `Sh.Nr`, `Sh.DB` and `Sh.BIO` are Shannon diversity indexes, and `Sc.Nr`, `Sc.DB` and `Sc.BIO` are Shade tolerance indexes. The shade tolerance indices calculated with different species abundance measures (number of canopy trees, biomass, and basal area) are highly correlated, with a Pearson's coefficient $r > 0.8$, and therefore only one of these variables can be employed.

4 Temporal analysis of the correlations

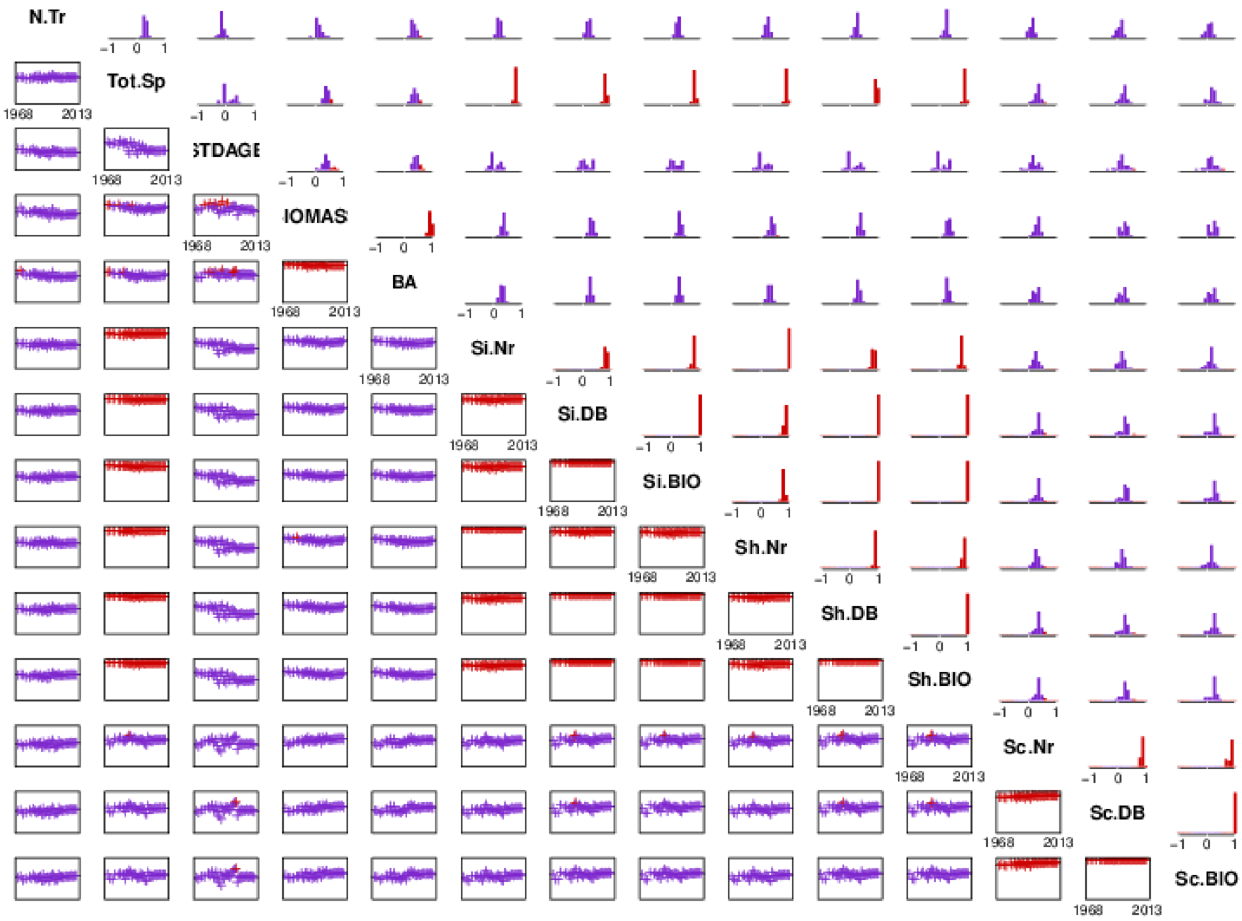

Figure 5: Correlations in the whole dataset broken down by years. The lower panel represents the yearly correlation coefficients as a function of time, with y-axis ranging from -1 to 1; the upper diagonal are histogram of the correlations represented in the lower diagonal. Years 1975, 1976 and 2013 were excluded as they contained less than 500 observations. Abbreviations are as in Figure 4.

5 Spatial analysis of the correlations

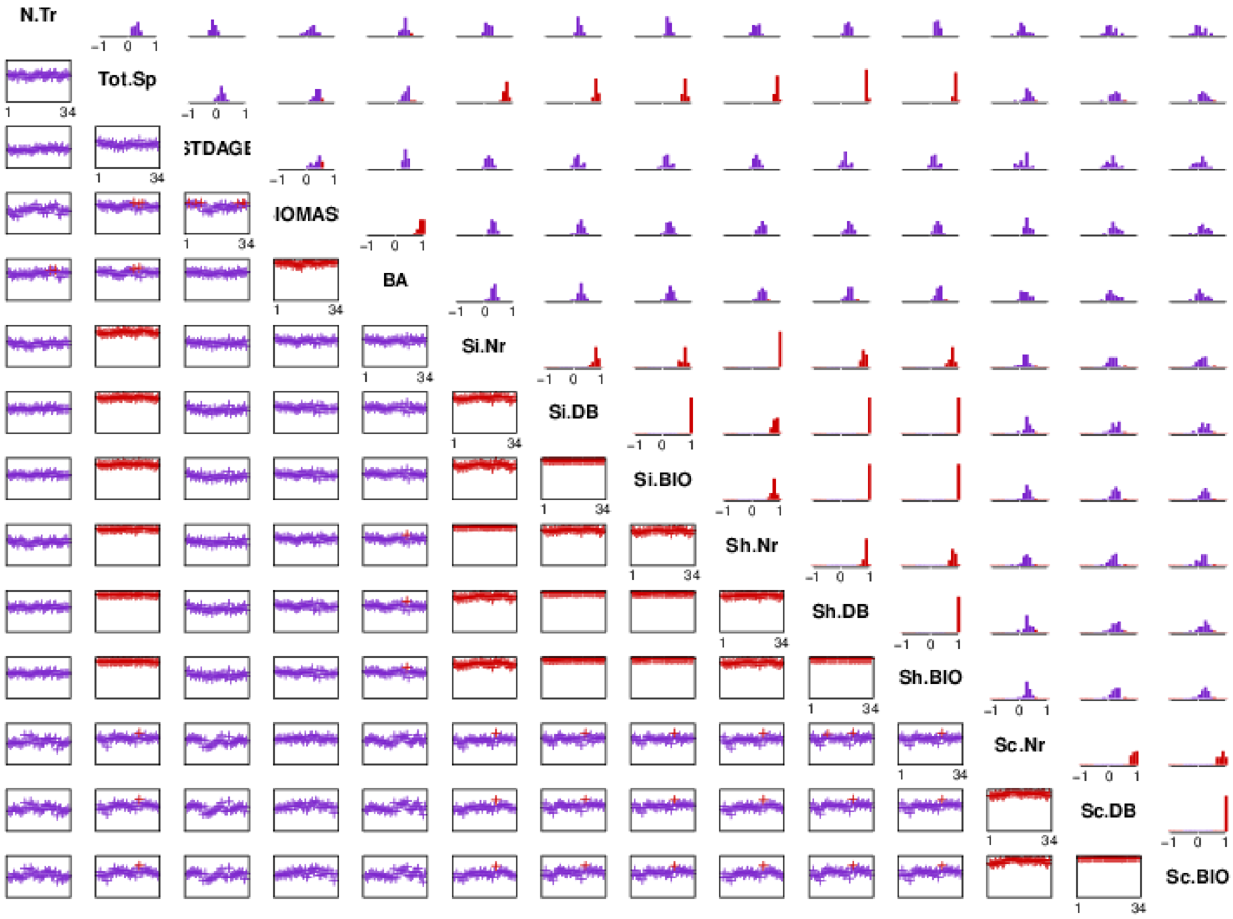

Figure 6: Correlations in the whole dataset broken down by ecoregion, similarly to Figure 5. Provinces 262 and 263 were excluded because they contained less than 500 observations. Abbreviations are as in Figure 4.

References

Bailey, R. G. (1995). *Description of the ecoregions of the United States.* Number 1391. US Department of Agriculture, Forest Service, 2 edition.

Friendly, M. (2002). Corrgrams: Exploratory displays for correlation matrices. *The American Statistician*, 56(4):316–324.
