# Supplementary material for: An Appraisal of the Classic Forest Succession Paradigm with the Shade Tolerance Index
Source: PLoS One. 2015 Feb 6;10(2):e0117138. doi: 10.1371/journal.pone.0117138 (PMC4319751; doi:10.1371/journal.pone.0117138)
Supplement: S5 Appendix — (PDF) [file pone.0117138.s005.pdf]

APPENDIX 5

Supplementary Results for the classification of the US Ecoregions

Supplement to the article "*An appraisal of the classic forest succession paradigm with the shade-tolerance index.*"

Jean Lienard [1], Ionut Florescu[2], Nikolay Strigul[1*],

[1]- Department of Mathematics & School of Art and Sciences, Washington State University Vancouver.

[2]- Financial Engineering Division and the Hanlon Financial Systems Lab, Stevens Institute of Technology, Hoboken, NJ, USA

[*]- nick.strigul@wsu.edu

1 Transition matrix estimation

1.1 Methodology

It is challenging to estimate the transition probabilities of shade tolerance in a Markov chain model based on forest inventory data, because the sampling interval of plots is highly irregular. To overcome this difficulty, we employed here the original methodology developed in Lienard et al. (2014). Specifically, we applied Gibbs sampling, a Monte Carlo Markov Chain implementation (Robert and Casella, 2004) to estimate the transition probabilities, with the specific guidelines provided by Pasanisi et al. (2012) (see also Gelfand and Smith, 1990). We provide in the following a brief description of the algorithm; please refer to Lienard et al. (2014) for an extended description.

The general idea is to use repeated measurements on the same plot to obtain the underlying transition matrix. Informally, we first discard all plots that were measured only once, and then align the measurements in sequences, independently of the year of measurement. These sequences are sparse, as the surveys in the database have be with irregular intervals. The core idea of our implementation of the Gibbs sampling algorithm is to estimate both the transition matrix and the missing values of these sequences. To do this, we perform two iterative steps. First, we sample compatible values for filling the missing measurements using a model of the matrix transition: this results in a model of the missing data. Second, we sample transition probabilities values to derive a new model of the matrix that are compatible with the modeled missing data.

More formally, we first constructed a temporal sequence S_p of the shade tolerance index for each plot p, by inserting the discretized value of shade tolerance index $s_{(p,i)}$ measured in the i-th year, at position i of S_p. Each temporal sequence S_p is mostly composed of unknown values, as only a fraction of the forest plots were surveyed each year. We then reduced the sparseness of these sequences by averaging the values in 3-year bins. We further extracted sub-sequences of length 5 (thus spanning 15 years) that fulfilled three criteria: (a) each subsequence starts with a known value, (b) each subsequence contains at least two known values. Let Y be the matrix constructed using all the sub-sequences, with rows corresponding to successive measures of different plots and columns corresponding to different time steps. The initialization of Gibbs sampling consists of replacing the missing values in Y at random, resulting in so-called augmented data $Z^{[0]}$. Then, the two following steps are iterated $H = 500$ times:

1. in the **parameter estimation** step, we draw a new transition matrix $\Phi_i^{[h]}$ conditional on the augmented data Z^{h-1}:

$$\Phi_i^{[h]} | Z^{[h-1]} \sim Dir(\gamma_{i,1} + w_{i,1}^{[h-1]}, ..., \gamma_{i,r} + w_{i,r}^{[h-1]}) \tag{1}$$

with Dir is the Dirichlet distribution, γ are biasing factors set here uniformly to 1 as we include no prior knowledge on the shape of the transition matrix (Pasanisi et al., 2012), and $w_{i,j}$ are the sufficient statistics defined as

$$w_{i,j} = \sum_{t \,\in\, \text{years}} \sum_{k \,\in\, \text{plots}} \mathbb{1}_{\{Z_{k,t-1}^{[h-1]} = s_i \;\&\; Z_{k,t}^{[h-1]} = s_j\}} \tag{2}$$

2. in the **data augmentation** step, we draw new values for the missing states:

$$\text{for the earliest data } t = 1, \quad \mathbb{P}(z_{k,1}^{[h]} = s_j | z_{k,2}^{[h-1]} = s_i, \Phi^{[h]}) \;\propto \Phi_{j,i}^{[h]} \tag{3}$$

$$\text{for the latest data } t = T, \quad \mathbb{P}(z_{k,T}^{[h]} = s_j | z_{k,T-1}^{[h]} = s_i, \Phi^{[h]}) \;\propto \Phi_{i,j}^{[h]} \tag{4}$$

$$\text{otherwise,} \quad \mathbb{P}(z_{k,t}^{[h]} = s_j | z_{k,t-1}^{[h]} = s_{i_1}, z_{k,t+1}^{[h-1]} = s_{i_2}, \Phi^{[h]}) \;\propto \Phi_{i_1,j}^{[h]} \times \Phi_{j,i_2}^{[h]} \tag{5}$$

We performed the whole procedure $R = 100$ times. We ignored the first $B = 100$ "burn-in" iterations, leaving $R \times (H - B) = 4000$ transition matrices for each ecoregion. The standard errors of the mean of the transition probabilities were consistently small, so we were finally able to derive the matrices presented in main text (as well as Figures 2 and 3 in this appendix) as the mean values.

1.2 Transition period

The precise choice of using 3-year transitions result from a tradeoff between:

- the need of individual-based models, which usually manipulate a 1-year transition period as their time step (e.g. Strigul et al., 2008).
- the usual time interval between successive of inventories is 5 years (as stated in the new unified FIA protocol — regional protocols before 1999 used different intervals).

The estimation method that is we relied on is suited to provide a precise estimate has been conceived specially for dealing with datasets having irregular measurement intervals (Lienard et al., 2014). Using this methodology, the choice of a rather short transition interval of 3 years does not substantially impact the underlying dynamics modeled; on the contrary, computing the 5-year transition matrices would force to discard all transitions over 3 years and 4 years observed in some older protocols - thus resulting in a less precise modeling of transitions. Also, multiplying the transition matrix by itself doubles the time steps - thus it is very easy to deduce transition matrices over longer intervals.

2 Shade tolerance classification across the US

Table 1: Shade tolerance indicators summarized for each US province.

Province	Plots	Resampled plots	Distribution width	Spectral gap
232	91856	60751	0.5	0.069
212	82401	36975	**0.9**	**0.023**
231	54649	35165	0.6	0.081
M221	23137	13696	0.7	0.060
223	24766	10578	**0.8**	**0.035**
221	20646	9491	**0.8**	**0.035**
222	23594	9462	**0.9**	**0.033**
211	14060	6003	**0.8**	**0.034**
M211	10345	4712	0.6	**0.047**
251	9855	3478	**0.8**	**0.040**
234	2605	1062	**0.8**	0.064
M231	2040	980	0.7	0.059
M223	1268	677	0.7	**0.047**
M331	5470	616	0.7	**0.032**
255	2846	542	0.7	0.085
332	1565	479	0.7	**0.052**
313	3499	452	0.4	0.058
M261	4755	374	0.7	**0.049**
341	2338	353	0.4	**0.046**
411	585	328	0.7	0.113
M341	2472	313	0.7	0.054
331	1819	295	0.7	**0.043**
M334	634	295	0.5	0.073
M242	5148	224	**0.9**	0.094
M313	2130	174	0.5	**0.052**
M332	4416	146	0.7	NA
321	1509	91	0.4	NA
322	470	65	0.4	NA
342	927	42	0.6	NA
263	418	34	0.6	NA
M333	3105	33	**0.9**	NA
M262	250	13	0.7	NA
242	544	13	**0.8**	NA
261	144	7	**0.8**	NA
262	5	0	0.5	NA
315	3597	0	**0.8**	NA

3 Dynamics of relative shade tolerance index

To investigate the dynamics of shade tolerance index in the early years of each area delimited based on the ecoregion classification, we first computed for each ecoregion the value of shade relatively to their first value (that is, the average value of shade tolerance index for stands that are one year old), and pooled together ecoregions belonging to the same area (with either "wide" or "narrow" distribution, and either "fast" or "slow" convergence). We further modeled the dynamics with a piecewise linear model with two segments, by fitting them to the formula:

$$y = \begin{cases} \alpha x & \text{if} \quad x < \mathrm{m} \\ \\ \alpha m + \beta x & \text{otherwise} \end{cases}$$

As we aligned the shade tolerance index to be 0 for the earliest stand age, this formula constraints the starting value $y = 0$. We performed the optimization using the Gauss-Newton algorithm, initialized with reasonable starting guesses (Bates and Watts, 1988). After convergence, all parameters α, m and β were found to be significantly different from 0 (one-sample two-sided t-tests, n between 7753 and 181718, $p < 0.001$). Due to the spread of the shade tolerance index, the fits were overall poor, with residual standard errors between and 0.135 and 0.260: while the established piecewise linear models are useful to highlight trends in the dataset, they should be used with caution when deriving quantitative predictions of shade tolerance index from the stand age.

The conceptual model predicts a decrease of the first stages followed by an increase. Specifically, ecoregions with a wide distribution of shade tolerance index exhibit the expected pattern regardless of their convergence speeds (bottom panels of Figure 1). Their computed inflection point, around 16-18 years, is further consistent with the study of the White Pine-Eastern Hemlock forests. Ecoregions characterized as having a narrow distribution and a slow convergence exhibits an early decrease of shade tolerance, however there is barely any following increase (top-right panel of Figure 1, the β slope is one order of magnitude lower than the other parameters). Finally, the area characterized by a narrow distribution and a fast convergence exhibit a pattern exactly opposite to what we expect in the shade tolerance driven succession, with an early increase followed by a decrease.

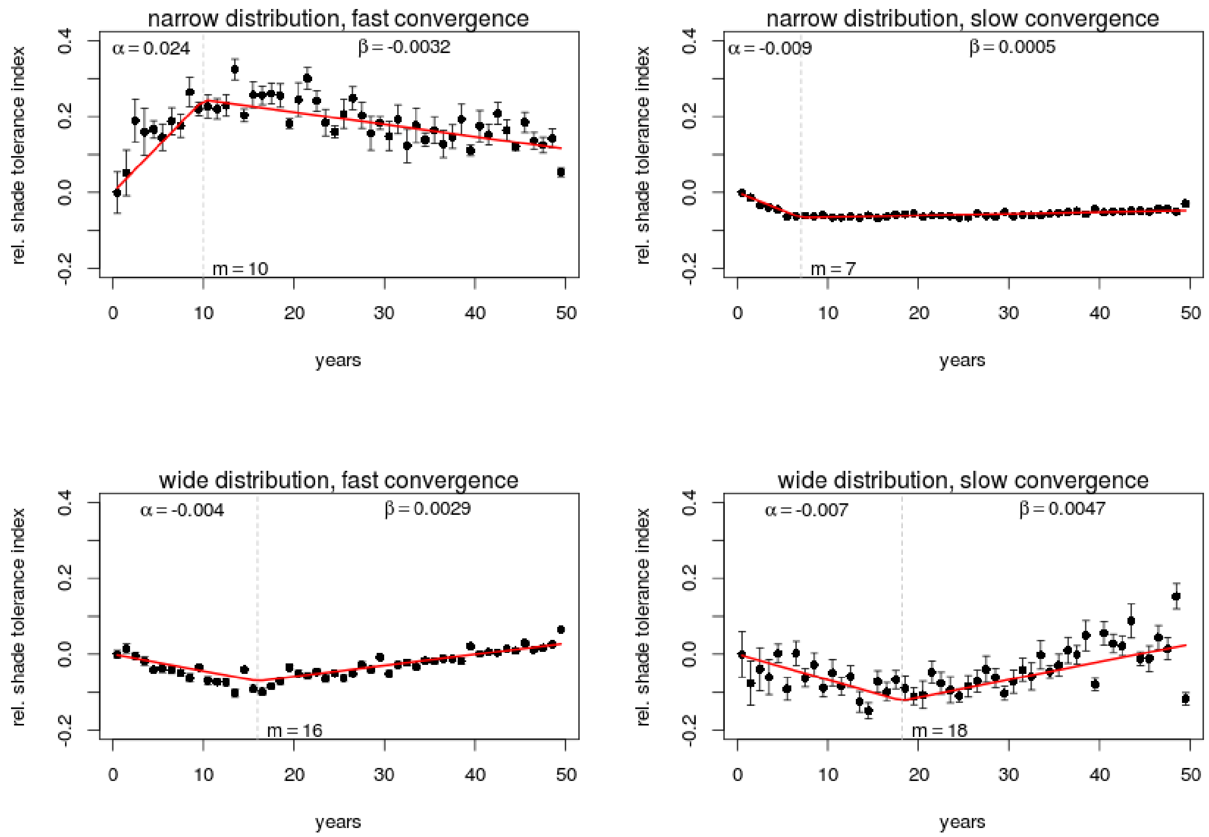

Figure 1: Relative shade tolerance index per area (in black, the error bars are standard errors of the mean) and piecewise linear regression (in red) with α being the slope of the first segment, β the slope of the second segment and m the age of the inflection point.

4 Transitions matrices computed on data subsets

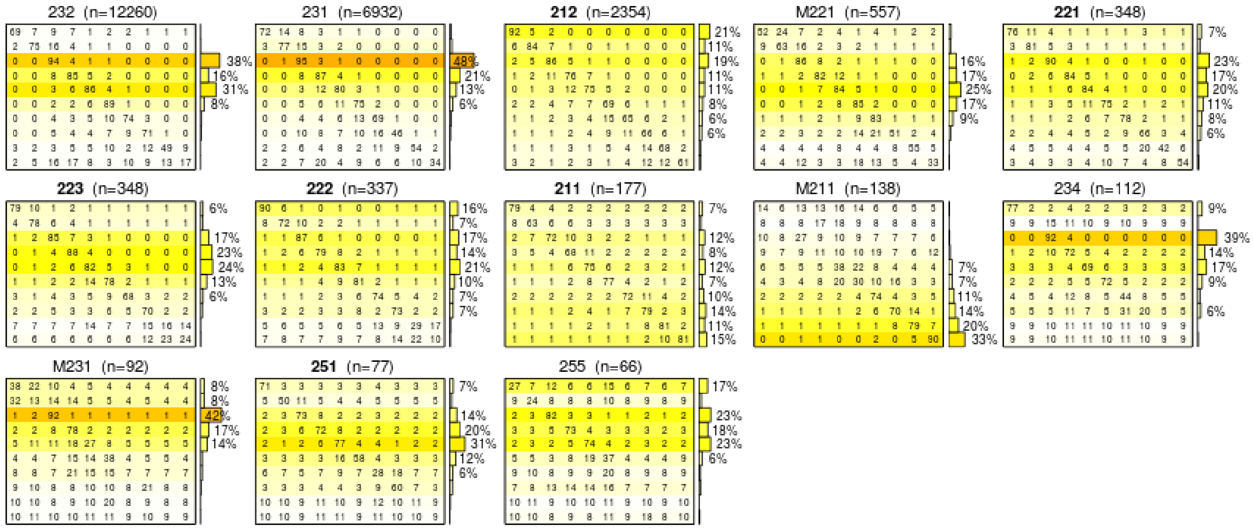

Figure 2: 3-year transition matrices computed on the subset of plots with a stand age less than 20 years. The transitions are obviously much more noisy than the ones obtained on the full dataset (in main text), which is expected due to the very limited number of re-sampled young plots. Except for ecoregion "M211", the early decrease of shade tolerance index predicted in the conceptual model is apparent in the matrices, with probabilities of decrease (transitions under the diagonal) superior to the probability of increase (transitions above the diagonal). The notations are similar to the ones in main text.

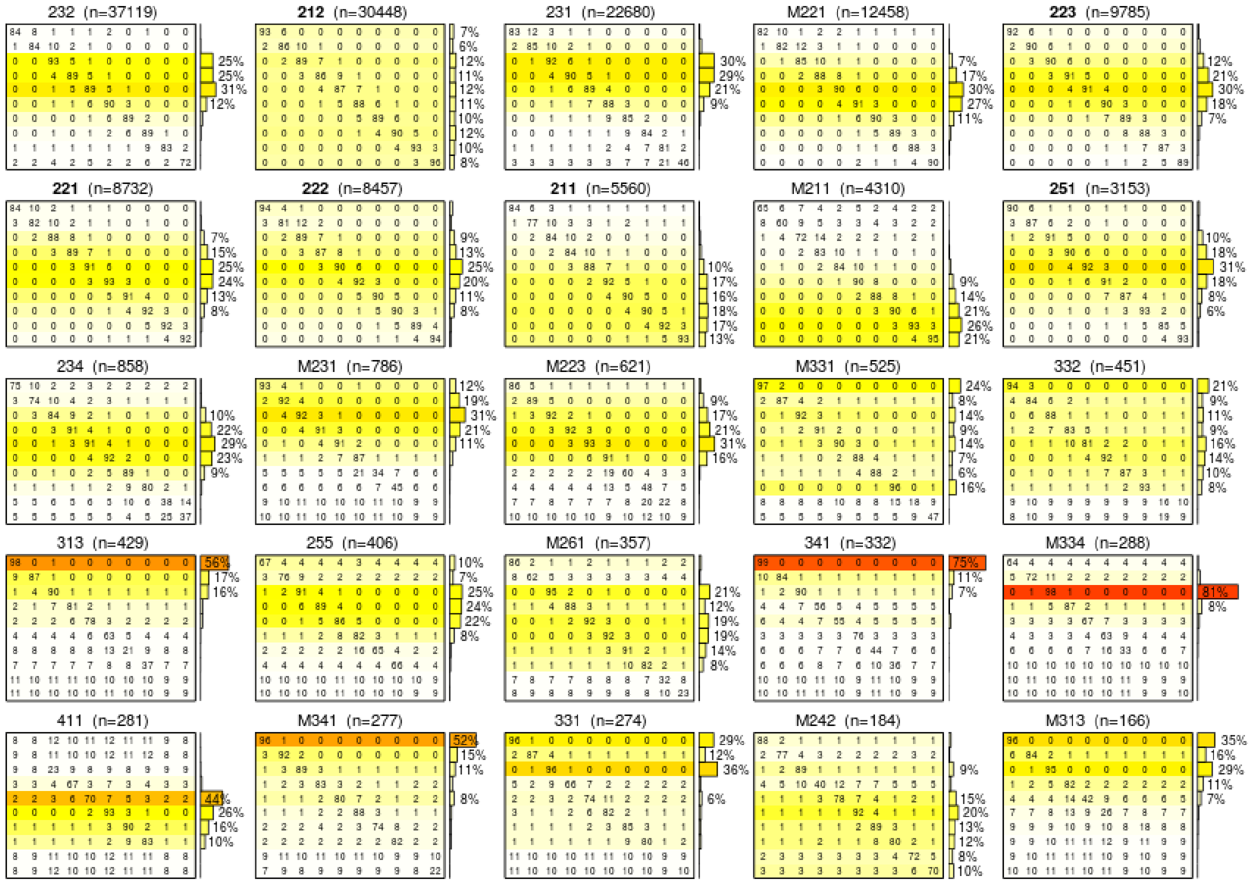

Figure 3: 3-year transition matrices computed on the subset of plots with a stand age more or equal to 20 years. The transitions mostly match the ones obtained on the full dataset (displayed in main text), confirming that transitions in the early stages (Figure 2 in this appendix) are not overall substantially contributing to the transition matrices. The notations are similar to the ones presented in main text.

References

Bates, D. M. and Watts, D. G. (1988). *Nonlinear regression: iterative estimation and linear approximations.* Wiley Online Library.

Gelfand, A. E. and Smith, A. F. M. (1990). Sampling-based approaches to calculating marginal densities. *Journal of the American statistical association*, 85(410):398–409.

Lienard, J., Gravel, D., and Strigul, N. S. (2014). Data-intensive multidimensional modeling of forest dynamics. *http://dx.doi.org/10.1101/005009.*

Pasanisi, A., Fu, S., and Bousquet, N. (2012). Estimating discrete markov models from various incomplete data schemes. *Computational Statistics & Data Analysis*, 56(9):2609–2625.

Robert, C. P. and Casella, G. (2004). *Monte Carlo statistical methods*, volume 319. Citeseer.

Strigul, N., Pristinski, D., Purves, D., Dushoff, J., and Pacala, S. (2008). Scaling from trees to forests: tractable macroscopic equations for forest dynamics. *Ecological Monographs*, 78(4):523–545.
